# The new general biological property of stem-like tumor cells Part I. Peculiarities of the process of the double-stranded DNA fragments internalization into stem-like tumor cells

**DOI:** 10.3389/fgene.2022.954395

**Published:** 2022-09-08

**Authors:** Genrikh S. Ritter, Evgeniya V. Dolgova, Daria D. Petrova, Yaroslav R. Efremov, Anastasia S. Proskurina, Ekaterina A. Potter, Vera S. Ruzanova, Svetlana S. Kirikovich, Evgeniy V. Levites, Oleg S. Taranov, Alexandr A. Ostanin, Elena R. Chernykh, Nikolay A. Kolchanov, Sergey S. Bogachev

**Affiliations:** ^1^ Institute of Cytology and Genetics of the Siberian Branch of the Russian Academy of Sciences, Novosibirsk, Russia; ^2^ Novosibirsk National Research State University, Novosibirsk, Russia; ^3^ State Research Center of Virology and Biotechnology “Vector”, Koltsovo, Russia; ^4^ Research Institute of Fundamental and Clinical Immunology, Novosibirsk, Russia

**Keywords:** carcinoma Krebs-2, Epstein-Barr virus–induced B-lymphoma, internalization of double-stranded DNA, heparin/dextran sulfate/polyanion-binding sites, clathrin/caveolar mechanism internalization

## Abstract

Stem-like tumor cells of ascites carcinoma Krebs-2 and Epstein-Barr virus–induced B-lymphoma were shown to possess the innate capability of binding and internalizing the TAMRA-labeled double-stranded DNA (dsDNA) probe. The process of binding and internalizing is rather complicated and composed of the following successive stages: 1) initiating electrostatic interaction and contact of a negatively charged dsDNA molecule with a positively charged molecule(s) on the surface of a stem-like tumor cell; 2) binding of the dsDNA probe to a tumor stem cell surface protein(s) *via* the formation of a strong chemical/molecular bond; and 3) the very internalization of dsDNA into the cell. Binding of DNA to cell surface proteins is determined by the presence of heparin/polyanion-binding sites within the protein structure, which can be competitively blocked by heparin and/or dextran sulfate, wherein heparin blocks only the binding, while dextran sulfate abrogates both binding and internalization. The abrogation of internalization by dextran sulfate implies the role of scavenger receptors in this process. Cells were shown to uptake DNA in amounts constituting ∼0.008% of the haploid genome. Inhibitors of caveolae-dependent internalization abrogate the DNA uptake in Krebs-2 cells, and inhibitors of the clathrin/caveolar mechanism block the internalization in B-lymphoma cells. In the present report, it is shown for the first time that in contrast to the majority of committed tumor cells, stem-like tumor cells of Krebs-2 and B-lymphoma carry a general positive charge on their surface.

## 1 Introduction

Previously, we have demonstrated that PCR-derived TAMRA-labeled double-stranded human *Alu* repeat is capable of being natively internalized by poorly differentiated cells of various origins, namely, hematopoietic stem cells (HSCs), mesenchymal stem cells, and tumor stem-like cells (TSCs) of different tumors, both experimental and obtained from clinic (Dolgova et al., 2014; 2016a, 2018, 2021) (Supplementary Material S1). The subpopulation of these cells varied from 0.01 to 7% for different cell “communities,” while the rest cells of these communities were incapable of internalizing extracellular TAMRA-labeled double-stranded DNA (dsDNA).

The stem-like status of TAMRA-positive cells in tumors and tumor cell cultures had been demonstrated both in direct grafting experiments for Krebs-2 murine carcinoma and Epstein-Barr virus–induced (EBV+) human B-lymphoma and in the comparative transcriptome analysis performed for their TAMRA+/TAMRA-counterparts (Potter et al., 2017; Supplementary Material S2). Elimination of TAMRA-positive cells from the tumor node results in experimental animals being cured of fulminant lethal forms of murine cancers (Ruzanova et al., 2022), and this is another piece of circumstantial evidence of the stem-like capabilities of these cells.

Discovering such fundamentally different properties of two types of cells within the same cell population inevitably raised the issue of molecular differences in the “architecture” of their cytoplasmic membrane. Moreover, experimental confirmation of this property of stem cells of various origins could become the basis for a novel paradigm describing their interplay with extracellular nucleic acids.

The surface of eukaryotic cells is commonly believed to bear a negative charge, determined by membrane-anchored glycoproteins carrying sialic acid residues. It is also known that DNA is a negatively charged molecule, and its association with negatively charged cell surfaces is prohibited due to *Coulomb* repulsion. This contradiction can be resolved only by the presumption that stem cells, unlike all other cell types, possess some property allowing them to interact with a negatively charged double-stranded DNA (dsDNA) molecule. We have presumed that this property is the positively charged cell surface/glycocalyx.

**FIGURE 1:**
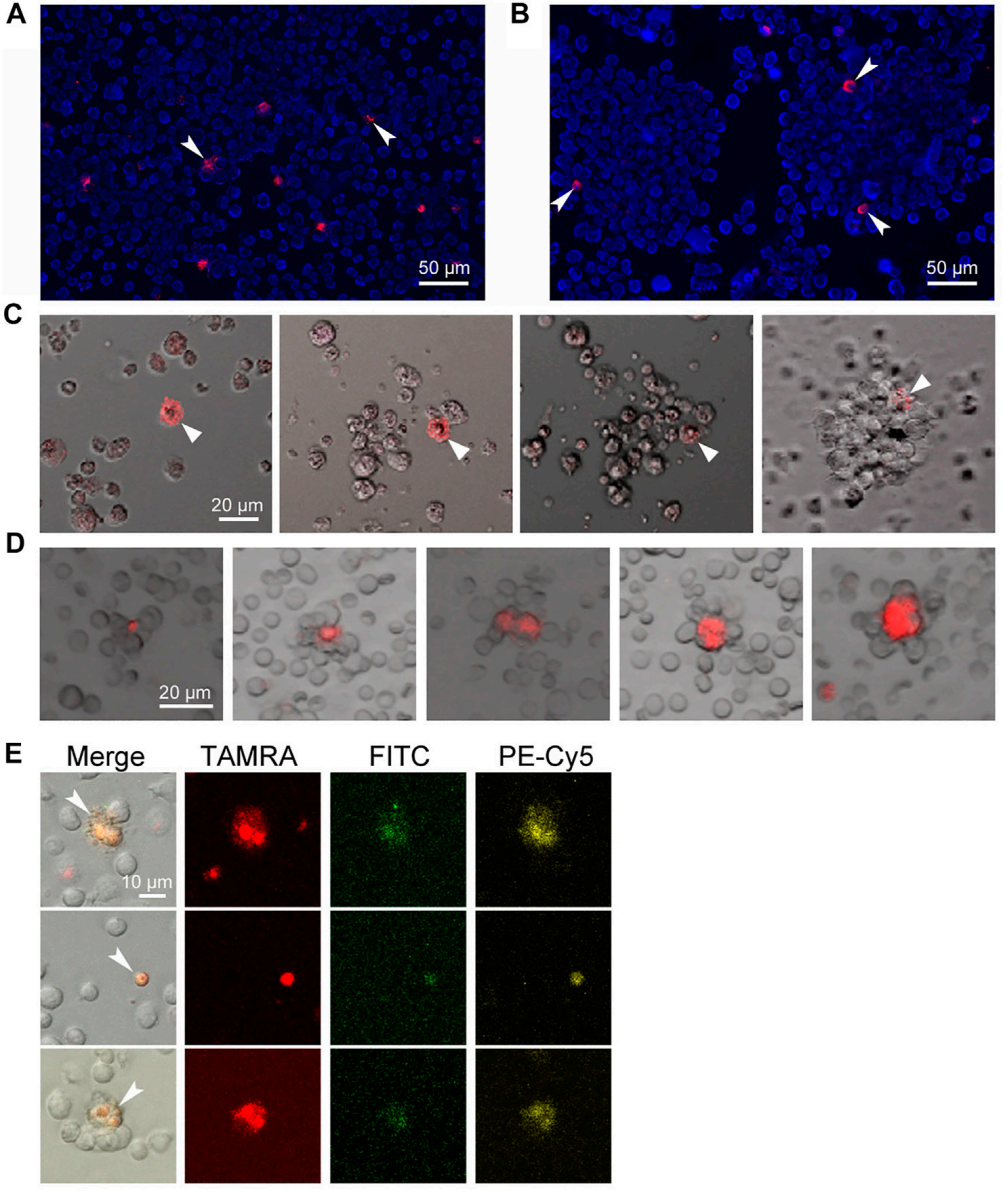
Internalization of the TAMRA-labeled DNA probe into stem cells of different genesis. Arrows denote the TAMRA + cells. **(A)** Krebs-2 cells. **(B)** Cells of Epstein-Barr virus–induced B-lymphoma. Two individual spheres are shown. **(C)** Assembling of the sphere in the culture of Epstein-Barr virus–induced B-lymphoma. Within 20–30 min, the TAMRA + cell is being associated with several proximal cells. During the whole time of observation, all cells in the field of view are in continuous chaotic movement. Upon the contact of TAMRA + cell with other cells, the strong aggregate is formed, providing the basis for further self-assembly of a free-floating sphere within 8–14 h. **(D)** Rosettes of bone marrow stromal niches with TAMRA + cells in their center. **(E)** The cells are both positive for their ability to internalize the TAMRA-labeled dsDNA and markers of pluripotent hematopoietic progenitors c-Kit (PE-Cy5) и Sca1 (FITC). The bone marrow was shown to contain 1.7% Sca1+ cells, 9.4% c-Kit + cells, 5.4% TAMRA + cells, 0.004% TAMRA+/Sca1+, 1.3% TAMRA+/c-Kit+, and 0.35% TAMRA+/c-Kit+/Sca1+ cells.

Stem cells are known to essentially depend on stromal cells, which, in association with stem cells, form stem niches, and glycocalyx plays an essential role in these structures (Santoro et al., 2016; Fuchs and Blau, 2020a). The assembly of spheres in the culture of Epstein-Barr virus-induced B-lymphoma starts with the formation of a sphere-initiating complex, consisting of a TAMRA + cell and several unstained cells, which occurs within 20 min and looks like the attraction of TAMRA + cells and their TAMRA– counterparts. Thorough examination of this process revealed no signs of active cell migration, such as pseudopodia formation and others, and thus might testify to the Coulomb attraction of differently charged cells (Figures 1A–C) (Dolgova et al., 2016b, 2019). Moreover, in cytological preparations of mouse bone marrow, structures constituted of cell aggregates with TAMRA+/c-Kit/Sca1/CD34+ (HSCs and their multipotent progeny with internalized TAMRA-labeled dsDNA probe) cells in their center were always observed (Dolgova et al., 2013; Ritter et al., 2020) (Figures 1D,E). Such a structure could be a result of the stem niche preformation, during which the stem cell attracts and fixes stromal cells around itself. It has been presumed that the physical basis for the initial contact between the stem cell and the recruited stromal ones is the positive charge of the stem cell membrane and the negative charge of the stromal cells. If the concept is correct, then such an interaction represents an effective and energy-independent mechanism ensuring the initial recruitment of stromal cells to the forming stem niche. As soon as the primary niche/sphere-initiating center is formed, the stem cell fixes already recruited stromal cells and recruits additional ones by secreting the appropriate chemokines and expressing the essential glycocalyx components (Dolgova et al., 2016b).

In the present study, the hypothesis of dsDNA binding/internalization by stem cells, implying the electrostatic attraction between the positively charged poorly differentiated cell and the negatively charged dsDNA molecule as the initial stage, has been experimentally tested. The remaining details of the mechanism have been determined in further experiments.

## 2 Materials and methods

### 2.1 Mouse line

We used CBA/Lac mice housed under non-SPF conditions (mouse-keeping facility at the Institute of Cytology and Genetics of the Siberian Branch of the Russian Academy of Sciences (IC&G SB RAS)). Mice were held in groups of 5–10 animals in plastic cages installed in standard mouse housing rooms with free access to food pellets (PK120-1, Laboratorsnab, Moscow, Russia) and water.

The study was conducted in accordance with the Declaration of Helsinki and approved by the Animal Care and Use Committee of the IC&G SB RAS (protocol No. 8 dated 19 March 2019).

### 2.2 Tumor models

Murine Krebs-2 carcinoma cell line was provided by the cell line repository of the IC&G SB RAS. This cancer cell line is maintained in mice as a transplantable tumor. To obtain ascites-bearing mice, 2 × 10^6^ of Krebs-2 cells in 200 µl of normal saline were inoculated intraperitonially. Ascites growth was monitored by weighing the animals.

To prepare the suspension, ascitic fluid was sampled from the peritoneum using an insulin syringe, placed into RPMI-1640 medium (Biolot, Russia), and sedimented at 400 g for 5 min. The cell pellet was then resuspended in RPMI-1640 medium again, and the cells were counted in the Goryaev chamber for further use.

Human Epstein-Barr virus–induced B-lymphoma cell line (EBV + B-lymphoma), derived from the aspirate of bone marrow cells of a patient diagnosed with multiple myeloma, was obtained from the Research Institute of Fundamental and Clinical Immunology (Novosibirsk, Russia) (Dolgova et al., 2019). The cells were cultured in Dulbecco’s Modified Eagle’s Medium (DMEM, Gibco, United States) supplemented with 40 μg/ml gentamycin sulfate and 10% fetal bovine serum (FBS) (HyClone, Logan, Utah, United States) at 37°C and 5% CO_2_ in a CO_2_ incubator (Memmert, United States).

To prepare the suspension, cells were gently detached from the plastic by pipetting and sedimented at 400 g for 5 min. The cell pellet was then resuspended in DMEM.

### 2.3 Treatment of cells with the TAMRA-dsDNA probe

As a TAMRA-dsDNA probe, the human Alu repeat labeled with TAMRA-5′-dUTP (Biosan, Russia) using PCR was used (Dolgova et al., 2014). 5 × 10^5^ cells were exposed to 0.1 μg of TAMRA-dsDNA in 500 μl of appropriate medium without additives for 30 min in the dark at room temperature. Cells were then subjected to a single washing with the appropriate medium and resuspended in the desired volume of the medium or phosphate-buffered saline (PBS) for further assays.

### 2.4 Electrophoresis of cells in the dialysis bag

10^6^ cells in 1 ml of PBS solution were transferred into a dialysis bag. The hermetically sealed bag was placed into the electrophoresis chamber filled with PBS that was used as a buffer. The contact of the bag with the buffer was accessed using paper bridges. Electrophoresis was conducted for 30 min at 4 V/cm. As soon as the electrophoresis was over, the bag was accurately clipped in the middle and cells from both the «+» and «–» parts were collected and sedimented at 400 g for 5 min. The cell pellets were resuspended in 500 µl of saline solution, and each fraction was exposed to the TAMRA-dsDNA probe. The content of TAMRA + cells was determined by both FACS assay and fluorescence microscopy.

1–2 × 10^5^ cells were cytospinned onto a glass slide, applied with DAPI/DABCO (0.5 μg/ml) (Sigma-Aldrich, United States), and covered with a coverslip. The preparations were examined using an AxioImager.M1 (Carl Zeiss, Germany) at the Collective Use Facility for Microscopic Analysis of Biological Objects, Siberian Branch of the Russian Academy of Sciences. TAMRA + cells were counted in accordance with the Order of the Ministry of Health of the Russian Federation dated 21 March 2003, “Improvement of anti-tuberculosis measures in the Russian Federation,” paragraph #11.4 “Microscopic examination procedure.” For each sample, ∼9,000 cells were assayed.

3 × 10^5^ cells were subjected to FACS assay using BD FACSAria III (BDBioscience, United States) at the Collective Use Facility for Flow Cytometry IC&G SB RAS. Four independent measurements were performed for Krebs-2 and six ones for EBV + B-lymphoma.

### 2.5 Treatment of cells with Basic Blue 41 cationic dye

5 × 10^5^ cells in 500 µl of saline solution were exposed to 2.7 μg/ml of Basic Blue 41 (BB41) (Sigma-Aldrich, United States) for 20 min at room temperature. Depending on the type of experiment, cells were exposed to TAMRA-dsDNA either before or after dye treatment. The content of BB41+ and TAMRA + cells was assessed using FACS assay. Three independent experiments with Krebs-2 and four ones with EBV + B-lymphoma were conducted.

### 2.6 Agarose micro-gel-electrophoresis

Cells exposed to the *Alu*-TAMRA dsDNA probe were washed once with saline solution, sedimented, and again resuspended in the desired volume of saline solution. 2 × 10^5^ cells were cytospinned onto a glass slide, applied with 20 μl of 2% low-melting agarose containing antifade DABCO (Sigma-Aldrich, United States) with DAPI (0.5 μg/ml) (Sigma-Aldrich, United States), and covered with a coverslip. As soon as agarose polymerized, the preparation was placed into the electrophoresis chamber filled with PBS that was used as a phoresis buffer. The contact of the preparation with the buffer was accessed using paper bridges. Electrophoresis was conducted for 30 min at 4 V/cm. Cytological preparations were examined using an AxioImager.M1 fluorescence microscope (Carl Zeiss, Germany).

### 2.7 Treatment of cells with EDTA and Ca++

5 × 10^5^ cells in 500 µl of PBS were subsequently exposed to 0.1 mM EDTA and 0.5 mM CaCl_2_ for 15 min each with a thorough double washing with PBS after each treatment. Treated cells were further exposed to the TAMRA-dsDNA probe. Next, the percentage of TAMRA + cells in samples was determined using BD FACSAria III (BDBioscience, United States). Three independent measurements were performed for every treatment regimen.

### 2.8 Treatment of cells with proteolytic enzymes

Cells were exposed to either 0.25% Trypsine-EDTA (Gibco, United States), 0.05% collagenase (Gibco, United States), or 0.002% of proteinase K (PrK) (Bioron, Germany) for 30 min at 37°С. Treated cells were further exposed to the TAMRA-dsDNA probe. Cells were either analyzed using FACSAria III (BDBioscience, United States) (four independent measurements were performed for every treatment regimen) or placed into a 12-well plate and analyzed using an LSM 780 NLO (Zeiss, Jena, Germany) fluorescent confocal microscope with ZEN software (Zeiss) (five independent measurements were done).

### 2.9 Treatment of cells with endocytosis inhibitors

Cells were exposed to the inhibiting compounds (sodium azide, EIPA, Cytochalasin D, Methyl-b-cyclodextrin, Nystatin, Dynasore, Wortmannin, Chlorpromazine, Dextran (Sigma-Aldrich, United States)), used in concentrations given in appropriate figures, for 30 min at 37°С. Treated cells were further washed and exposed to the TAMRA-dsDNA probe. The content of TAMRA + cells was analyzed using BD FACSAria III (BDBioscience, United States). The viability of cells was additionally monitored by checking the specificity of a signal during the fluorescent microscopy. Upon death or ongoing apoptosis, cellular autofluorescence increases in all channels, TAMRA, FITC, and DAPI. In our experiments on assessing the fluorescence, we utilize this feature for distinguishing the specific signal(s). True TAMRA-positive cells should fluoresce in the TAMRA-specific channel and should not in all others; otherwise, it is considered an artifact or corrupted cell. Three to six independent measurements were performed for each compound tested.

### 2.10 Real-time PCR-mediated detection of the internalized TAMRA-dsDNA probe

Cells were treated with endocytosis inhibitors used in the most effective concentrations and washed and exposed to the TAMRA-dsDNA probe. After this, cells were washed and incubated with 10 mM MgCl_2_ and 10 μg/ml of DNase for 10 min at 37°C. Furthermore, cells were washed twice and incubated with 20 μg/ml of PrK for 30 min at 37°C. Treated cells were washed twice and placed in lysis buffer (1% SDS, 50 mM EDTA, 20 μg/ml of PrK) for 60 min at 65°C with the following extraction of DNA with phenol/chloroform. Precipitated DNA was further dissolved in 10 mM TrisHCl, pH 8.0. Total amount of DNA in samples was assessed on a QUBIT fluorimeter (Thermo Fisher Scientific).

Quantitative PCR was performed using the iQ5 Multicolor Real-Time PCR Detection System (Bio-Rad) with SYBR Green fluorescent dye (quantitative PCRmix-HS SYBR, Evrogen, Russia) and Bio-Rad iQ5 software. Isolated DNA was used as a matrix in real-time PCR with primers specific to the used DNA probe (M13 for 5′-GTA​AA-ACG​AC-GGC​CA-G-3′, M13 rev 5′-CAG​GA-AAC​AG-CTA​TG-AC-3′) and to house-keeping genes (*Actb* in the case of Krebs-2: for 5′-GGT​GT-GAT​GG-TGG​GA-GAA​GC-3′, rev 5′-TCT​CC-ATG​TC-GTC​CC-AGT​TG-G-3′, and *Rplp0* in the case of EBV + B-lymphoma: for 5′-AGG​CC-TTC​TT-GGC​TG-ATC​CA-TCT-3′, rev 5′- TAT​CC-TCG​TC-CGA​CT-CCT​CC-GA-3′). Three rounds of real-time PCR experiments were performed with three samples each. Conversion of real-time PCR data into dsDNA copy numbers was made exactly as described previously (Dolgova et al., 2019).

### 2.11 Statistical processing of data

For statistical data processing, Statistica package version 10 (StatSoft, Tulsa, OK, United States) was used. Statistical routines used for each particular experiment are given in the figure legends. In the case of *a posteriori* comparisons, the confidence was estimated with Kruskal–Wallis ANOVA test (nonparametric test used for comparing multiple independent groups). Pairwise comparison of two independent samples was estimated with nonparametric Mann–Whitney *U*-test.

## 3 Results

### 3.1 The used models and terms

Two eukaryotic cell models, *in vivo* ascitic carcinoma Krebs-2 and *ex vivo* sphere-forming Epstein-Barr virus–induced B-lymphoma (EBV + B-lymphoma), were used. As a double-stranded DNA (dsDNA) probe, the PCR-derived TAMRA-labeled human *Alu* repeat was used. TAMRA + cells were previously shown to be tumor-initiating cells of Krebs-2 carcinoma (Dolgova et al., 2014). In the case of EBV + B-lymphoma, these cells are clonotypic clonogenic cells, which express the CD133 marker of cancer stem cells and are crucial for the development of subcutaneous grafts (Dolgova et al., 2016b, 2019). These data, as well as the results of transcriptome analysis and determining the clonogenic properties of TAMRA + cells of EBV + B-lymphoma suppose these cells to be tumorigenic stem-like ones (Petrova et al., 2022). Thus, in the current investigation, the interaction of two types of tumor stem cells with the TAMRA-labeled dsDNA probe has been analyzed.

The physical interaction of the probe with a cell, regardless of its intra- or extracellular localization, which results in specific fluorescence of the cell allowing this cell to be distinguished from the bulk of unstained ones, is referred to as “binding”.

The term “internalization” is reserved to the process resulting in intracellular localization of the probe confirmed by the confocal microscopy and/or real-time PCR.

The “interaction” comprises both the aforesaid processes without specifying details.

### 3.2 Characterization of the principles of the TAMRA–dsDNA probe interaction with Krebs-2 carcinoma and EBV + B-lymphoma cells

#### 3.2.1 Determining the charge of Krebs-2 and EBV + B-lymphoma cells, which interact with TAMRA-labeled dsDNA fragments

To determine the principles of the process of dsDNA probe interaction with cell surface components, the following were conducted:

• a series of electrophoretic separations in a free volume and
• estimation of the charge of cells, which bind the dsDNA probe, using the positively charged dye.

##### 3.2.1.1 Electrophoretic separation of cells in a free volume with the following estimation of the number of cells capable of binding TAMRA-dsDNA

We have developed an approach to estimate the charge of cells using an electrophoretic separation in a free volume of physiological saline solution. The use of alternative electrophoretic media (TAE, TBE, or PB) resulted in degenerative changes in cells and their osmotic destruction. The core of the approach boiled down to the following. Cells were placed into a dialysis bag, which was then hermetically sealed on both sides. The bag was overlaid with 3 MM Whatman paper bridges on both sides and subjected to the electrophoresis for not more than 40 min. A partial relocation of differently charged cells occurred. In the pilot experiments, erythrocytes were shown to move toward the “+” electrode. Additional experiments have indicated that an effective separation of cells is possible only upon a certain ratio of the medium volume to the number of cells, which was not more than 10^6^ cells per 1 ml of the carrier. An excess of cells resulted in disturbances, which hampered their free movement in the appropriate direction. After separation, cells were taken from their location within the dialysis bag, treated with TAMRA-dsDNA, and the number of TAMRA + cells was estimated by microscopy and/or flow cytometry assay.

**FIGURE 2:**
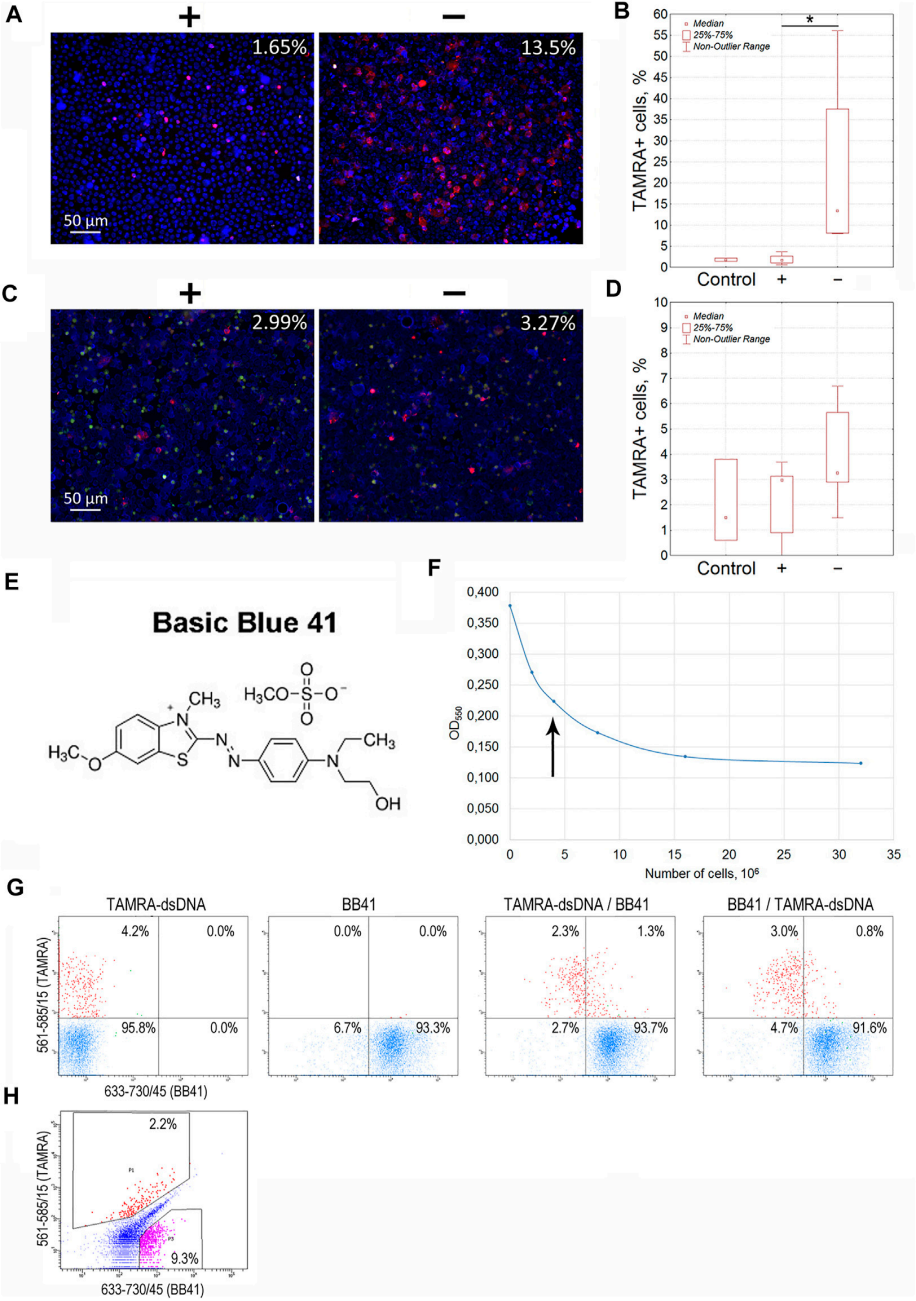
Determination of the charge of Krebs-2 carcinoma and EBV + B-lymphoma cells capable of interacting with the TAMRA-labeled dsDNA probe. **(A)** Microscopic images of Krebs-2 cells taken from the opposite “plus” and “minus” sides of the electrophoretic chamber (hermetically sealed dialysis bag) and exposed to TAMRA-dsDNA. In the upper right corner, the percentage of cells with the bound DNA probe is indicated (Median). **(B)** Counting the TAMRA + cells in fractions of Krebs-2 cells taken from the opposite sides of the electrophoretic chamber after separation (n = 4). **(C)** Microscopic images of EBV + B-lymphoma cells taken from the opposite “plus” and “minus” sides of the electrophoretic chamber and exposed to TAMRA-dsDNA. In the upper right corner, the percentage of cells with the bound DNA probe is indicated (Median). **(D)** Counting the TAMRA + cells in fractions of EBV + B-lymphoma cells taken from the opposite sides of the electrophoretic chamber after separation (n = 6). **(E)** The chemical formula of Basic Blue GRL 41. **(F)** Saturation curve showing the interaction of Krebs-2 cells with the dye. The arrow denotes the number of cells required for the saturation at the selected dye concentration used in experiments on the negative cell charge negation. **(G)** Flow cytometry assay of Krebs-2 cells sequentially exposed to TAMRA-dsDNA and Basic Blue GRL 41. **(H)** Flow cytometry assay of EBV + B-lymphoma cells simultaneously exposed to TAMRA-DNA and Basic Blue GRL 41. *—reliable differences, р<0.05 according to the Mann-Whitney *U*-test.

For Krebs-2 cells, the content of TAMRA + cells turned out to be always higher in the “–” area of the dialysis bag than in the “+” area (13.5 vs. 1.65%, *p* < 0.05), indicating these cells to be positively charged (Figure 2A,B). However, the total relocation of positively charged cells toward the negative electrode has not been achieved. Theoretical aspects of the electrophoretic separation of cells in a free volume are the subject of colloidal chemistry (Golovanov, 1998; Evstrapov, 2005; Liang et al., 2014) and thus remain beyond the topic discussed in this report, which is focused exclusively on the analysis of the results obtained. Nevertheless, we provide some hypothetical presumptions, which could explain the observed impossibility of total separation of cells according to their charge.

The movement of molecules, or, as in the current case, cells, in the electric field depends on a number of parameters, including the charge magnitude, frictional force of the medium and its electrolytic properties, etc. Under these conditions, the charged object is surrounded by the cloud of counterions, the so-called solvation sphere, which reduces the electrophoretic mobility of the object due to the following. First, counterions negate, either partially or completely, the charge, causing the object to be less affected by the electric field. Second, having an opposite charge, the cloud of counterions itself is affected by the electric field and shifts in the opposite direction, slowing down the solvated object. Such a phenomenon is known as the electrophoretic friction. Third, the ions constituting the solvation sphere are in permanent thermodynamic movement that results in continuous replacement of one ion by another. This process is time consuming and thus causes temporary disturbances in the symmetry of the solvation sphere. As a result, the double electric layer behind the charged object stretches, slowing down its movement. This phenomenon is known as the relaxation effect. In this regard, any of such factors as a rapid negation of the cell surface charge, an increasing gradient of positive (Na+) and negative (Cl–) ions, a depletion of the electrolytic capabilities, and an increase in the local content of cells bearing the same positive or negative charge could hamper the complete separation of oppositely charged cells.

Similarly, experiments on the electrophoretic separation followed by the estimation of the TAMRA + cell content were conducted for EBV + B-lymphoma. The results obtained were somewhat contradictory (3.27 vs. 2.99%, no significant difference). In some cases, the content of TAMRA + cells was higher in the anodic fraction relative to the catodic one, but in several cases, there were no differences observed between the fractions that lowered the general confidence of the results (Figures 2C,D; Table 1). Nevertheless, the data obtained testified to the general positive charge of at least a certain fraction of TAMRA + cells of EBV + B-lymphoma that caused their shift to the anode (which was further confirmed by the experiments with proteinase K (PrK) treatment and agarose micro-gel-electrophoresis, described in the following sections).

**TABLE 1:** Content of TAMRA + cells, %.

	Experiment #	Control	“+” fraction	“–” fraction
PrK “–”	1	1.5	0.9	3.6
	2	3.8	3.7	5.7
PrK “+”	3	1.5	3.0	6.7
	4	0.5	0.0	1.4

##### 3.2.1.2 Estimating the binding of dsDNA probe using the specific positively charged dye Basic Blue GRL 41

To verify the previously obtained results on determining the positive charge of TAMRA + cells in the models of Krebs-2 and EBV + B-lymphoma, a number of experiments on negating the charge of cells using Basic Blue GRL 41 (cationic, positively charged dye (BB41)), followed by assessing the percentage of TAMRA + cells (Figures 2E–H), have been carried out. If the binding directly depends on the positive charge present on the cell surface, or is associated with another factor being specific to positively charged cells only, then the TAMRA-dsDNA probe distribution will remain unchanged upon changing the sequence of cells’ exposure to TAMRA-dsDNA and BB41, and TAMRA + cells will be either partially or completely outside the subpopulation of those stained with BB41. Otherwise, there should be a significant redistribution of TAMRA-stained and BB41-stained cell subpopulations. Overlapping subpopulations, in this case, could be explained by the mosaic pattern of the positive charge distribution over the cell surface, interspersed with negatively charged areas. The following results have been obtained.

The distribution of Krebs-2 cells between the two subpopulations remained almost unchanged (3.6 ± 1.2% of TAMRA + cells in case of TAMRA-dsDNA treatment at first vs. 3.8 ± 0.9% of TAMRA + cells in case of BB41 pre-treatment, Median±SD, n = 3, no significant differences) and that confirms the presumption that dsDNA interacts with cells bearing the positive surface charge and being unstained with BB41. These experiments also indicate that negating the negative charge with cationic dye does not result in abrogation of mechanisms of DNA binding to “+” cells. A partial overlap can be explained by the presence of positively charged components on the surface of negatively charged cells, which mediate their capability of binding a certain amount of dsDNA (Figure 2G).

Flow cytometry assay of EBV + B-lymphoma cells revealed the presence of a major subpopulation with definitely increased autofluorescence in all detection ranges. Despite the set gates quite clearly defining both TAMRA+ and BB41 + subpopulations, which form two independent and distinct clusters in the appropriate dot-plot quartiles, the general distribution of cells precludes reliable tracing of the changes caused by differential treatments with TAMRA and BB41 (Figure 2H). The presence of such an “autofluorescent” subpopulation in the culture of EBV + B-lymphoma indicated the complexity of this model, which was a probable cause of the problems with detecting the charge of EBV + B-lymphoma cells in experiments on electrophoretic separation in a free volume.

The analysis performed suggests the following. For Krebs-2, the first factor mediating the interaction of dsDNA with a cell is either the general positive charge of a cell or some molecular component present on the surface of “positive” cells only. For EBV + B-lymphoma, the results obtained in experiments on electrophoretic separation also do not contradict the proposed concept of positively charged TSCs interacting with the TAMRA-dsDNA probe.

#### 3.2.2 Changes in the spatial localization of the TAMRA-dsDNA probe bound by Krebs-2 and EBV + B-lymphoma cells after micro-gel-electrophoresis

We have designed an experiment based on the micro-gel-electrophoresis of cells. It has been presumed that the surface localization of the bound dsDNA probe should (or should not) change during the electrophoresis according to the type and character of its interaction with cell surface components. It would allow us to determine whether the binding of dsDNA depends on the charge of cells only or if it requires the presence of additional factors mediating the chemical/molecular interaction.

The following procedures were performed. Krebs-2 or EBV + B-lymphoma cells were exposed to the TAMRA-DNA probe for up to 5 and up to 60 min. Electrophoresis was conducted in either two stages of 15 min each with visual control after each step or one 30 min stage without intermediate control. The following results have been obtained.

**FIGURE 3:**
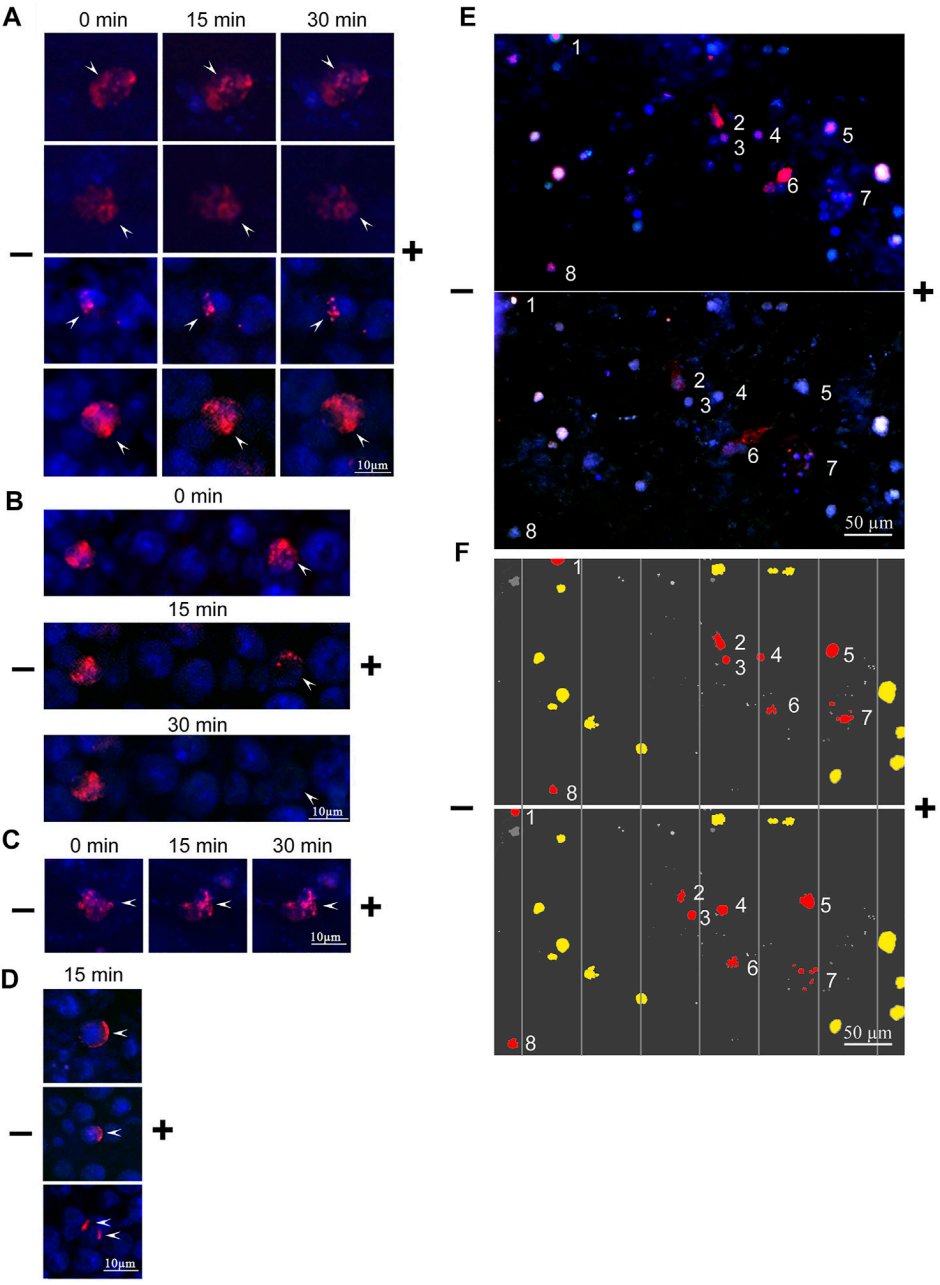
Changes in the spatial location of TAMRA-dsDNA, bound to Krebs-2 and EBV + B-lymphoma cells, after micro-gel-electrophoresis. Krebs-2 **(A–D)** and EBV + B-lymphoma **(E,F)** cells were incubated with the TAMRA-dsDNA probe, cytospinned, embedded into 1% low-melting agarose, and subjected to electrophoresis on a glass slide. The duration of electrophoresis is indicated atop the blocks. Arrows denote the TAMRA-labeled material. **(A)** TAMRA-dsDNA retains its spatial location during electrophoresis. The time of exposure to the probe is 40 min **(B)** TAMRA-dsDNA gradually exfoliates from the surface during electrophoresis (for demonstrative comparison, the cell with “immobile” fluorescent material is shown). The time of exposure to the probe is 3–5 min **(C)** TAMRA-dsDNA visually retains its spatial location during electrophoresis. The time of exposure to the probe is 3–5 min **(D)** TAMRA-dsDNA “drifts” across the membrane toward the anode. The time of exposure to the probe is 3–5 min. **(E)** Electrophoresis of EBV + B-lymphoma cells. **(F)** Digitally processed schematic image of EBV + B-lymphoma cell electrophoresis. The numbers denote the cells shifted toward the anode.

The mobility of the bound TAMRA-material along the Krebs-2 cell surface depended on the incubation time. In the majority of cells subjected to the long-term (60 min) exposure to TAMRA-dsDNA, the bound probe did not change its original location after electrophoresis was conducted (Figures 3A,B, left TAMRA + cell). For some cells, however, a complete “exfoliation” of the fluorescent material from the cell surface has been shown (Figure 3B, right TAMRA + cell). In the case of the short-term exposition, the shift of TAMRA-labeled material in the minus-to-plus direction has been observed in the significant majority of TAMRA + cells. This process was accompanied by the formation of a characteristic crescent pattern (Figures 3C,D). At the same time, in some cells, a portion of the bound fluorescent marker shifted, while the rest retained its initial intracellular location (Figure 3C).

To explain the results obtained, the following hypothesis has been proposed. Upon the long-term exposition, most of TAMRA-dsDNA is being internalized into the cell that results in its spatial stabilization and apparent resistance to the effect of the electric field. Upon the short-time exposition, a part of TAMRA-dsDNA remains on the outer side of the cell surface, while the remaining part is being internalized into the inner space of the cell. The first fraction shifts along the membrane in the minus-to-plus direction, forming the characteristic crescent, while the second, internalized one does not change its location. The shift of the membrane-bound fraction of dsDNA presumes its chemical/molecular bond to a certain cell membrane component capable of free drifting across the lipid layer (glycocalyx components and cholesterol rafts).

In the preparations, there were observed cells, in which the bound TAMRA-dsDNA did not form a strong bond to the membrane components and, as a result, completely exfoliated during electrophoresis. Referring to our previously reported data (Dolgova et al., 2016a), it can be presumed that these cells are in the G2/M phase of the cell cycle and, thus, have their internalization machinery inactivated due to the ongoing mitotic process. In this case, binding is determined exclusively by the electrostatic forces (as the first phase of internalization) without interaction with specific sites mediating the strong immobilization on the cell surface followed by the internalization.

The same approach was used in the case of EBV + B-lymphoma cells. TAMRA + cells were clearly shown to drift toward the negative electrode, presuming their positive overall charge (Figures 3E,F). Exfoliation of the TAMRA-labeled material during electrophoresis has been confirmed for some part of the cells. The result suggests that some fraction of the cells have the fluorescent material inside, thus keeping the external positive charge unaffected. For another fraction, the labeled material dissipates during electrophoresis, but cells, nevertheless, drift toward the anode (these cells are assumed to undergo the mitotic division and either lack the surface factors mediating strong dsDNA binding, or these factors are inaccessible to the ligand). Some TAMRA + cells do not shift during electrophoresis, which may indicate them to be dead. Some fraction of TAMRA–cells also shifts toward the anode, and this might be the consequence of the inaccessibility of polyanion-binding factors to their ligands as well.

The result obtained has determined the second factor of the interaction of the extracellular dsDNA probe with a cell. It is a chemical/molecular interaction with elements of the cell surface. Variants of weak association of the labeled probe with the Krebs-2 cell surface, which results in dissipation of the bound probe in the electric field, have been found.

For EBV + B-lymphoma, the results of electrophoresis in a free volume, indicating the positive charge of the majority of TAMRA + cells, have been confirmed. At the same time, some fraction of TAMRA + cells does not shift. There was also found a variant of the weak association of the labeled probe with the surface of B-lymphoma cells, which results in exfoliation of the bound probe due to the electric field application (Figures 3E,F, cluster of cells No 6).

#### 3.2.3 Changes in the percentage of TAMRA + cells after heparin pretreatment

**FIGURE 4:**
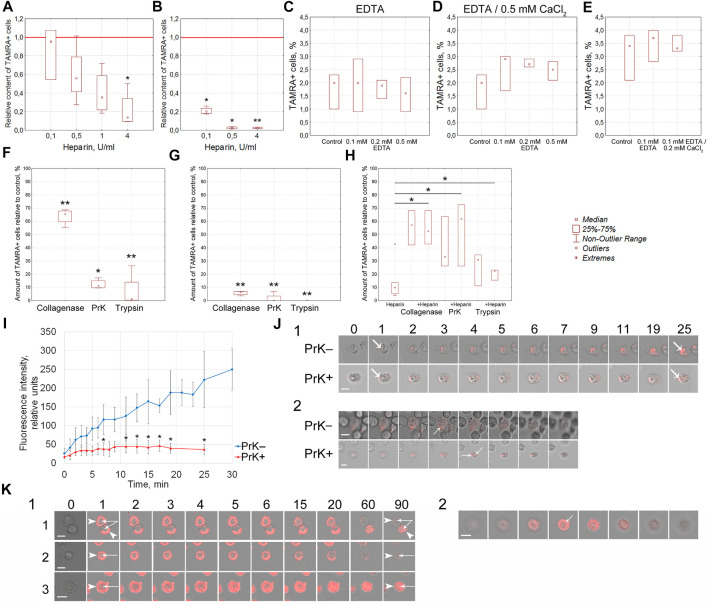
Molecular characterization of the surface elements of TAMRA + cells, determining the interaction of cells with the TAMRA-dsDNA probe. **(A)** Effect of heparin on the interaction of Krebs-2 cells with TAMRA-dsDNA (n = 4). **(B)** Effect of heparin on the interaction of EBV + B-lymphoma cells with TAMRA-dsDNA (n = 4). **(C)** Effect of EDTA at various concentrations on the interaction of Krebs-2 cells with TAMRA-dsDNA (n = 3). **(D)** Effect of EDTA at various concentrations with following exposure to СаCl_2_ on the interaction of Krebs-2 cells with TAMRA-dsDNA (n = 3). **(E)** Effect of EDTA at various concentrations with following exposure to СаCl_2_ on the interaction of EBV + B-lymphoma cells with TAMRA-dsDNA (n = 3). **(F–H)** Effect of collagenase, PrK, and trypsin on the interaction of Krebs-2 cells with TAMRA-dsDNA. The percentage of TAMRA + cells relative to the control (intact Krebs-2 cells incubated with TAMRA-dsDNA only, accepted as 100%) is shown. The content of TAMRA + cells has been assessed using FACSAria III cell sorter (BD, United States). **(F)** Cells were exposed to the appropriate enzyme for 10 min and then incubated with the TAMRA-labeled dsDNA probe (n = 4). **(G)** Cells were preincubated with the TAMRA-labeled dsDNA probe and then exposed to the appropriate enzyme for 10 min (n = 4). **(H)** Cells were pre-exposed to a certain proteolytic enzyme for 30 min, and then some samples were added with heparin, after which the samples were incubated with the TAMRA-labeled dsDNA probe (n = 3). **(I)** The curve of accumulation of the TAMRA-labeled dsDNA probe in intact cells and cells treated with PrK for 30 min. Average ±SD are shown (n = 5). **(J)** 1—the set of images demonstrating the results shown in the previous plot **(I)**. Numbers indicate the time of incubation with the TAMRA-labeled dsDNA probe, in minutes. 2—confocal “slices” of individual cells at the terminal stage of incubation, demonstrating the intracellular localization of the TAMRA-labeled dsDNA probe. **(K)** 1—Patterns indicating the fate of TAMRA-labeled material in Krebs-2 cells exposed to PrK. 1—2 cells with different fates of the labeled material; 2—the cell with the labeled material completely exfoliated after long-term exposure to PrK; 3—the cell subjected to long-term exposure to PrK with the labeled material detected in intracellular compartments. The time of incubation with TAMRA-dsDNA in minutes is shown atop images. Arrows—reliable differences relative to the control or between groups as indicated by the line, *p<0.05, **p < 0.01 according to the Kruskal–Wallis ANOVA test. Bars correspond to 10 µm.

Previously, we have reported that heparin blocks the binding of the TAMRA-dsDNA probe to Krebs-2 cells (Dolgova et al., 2016a). These data could indicate the competition between heparin and dsDNA for the same cell surface components. An efficacy of the TAMRA-dsDNA probe binding to cells pretreated with heparin has been estimated. Heparin in the amount of 4 U was shown to prevent the binding of the TAMRA-dsDNA probe to Krebs-2 cells (*p* < 0.05) and, in the amounts of 0.1, 0.5 (*p* < 0.05), and 4 U (*p* < 0.01), to EBV + B-lymphoma cells (Figures 4A,B). These results proved that the interaction of heparin and dsDNA with the cell surface is determined by the same factors in both cell models.

Numerous investigations over the past 20 years demonstrated that heparin binds to a variety of cellular and extracellular proteins (Muñoz and Linhardt, 2004; Ori et al., 2008; Torrent et al., 2012; Meneghetti et al., 2015; Ishihara et al., 2019). A long-time search for the specific heparin-binding site resulted in the conclusion that in the vast majority of cases, such a site is just a cluster of positively charged aminoacids, which form ion pairs with spatially separated negatively charged carboxyl and sulfate groups of glucoseaminoglycan. In many proteins, such clusters are grouped within the so-called collagen-like domain (Doi et al., 1993; Deprez and Inestrosa, 1995; Delacoux et al., 2000; Muñoz and Linhardt, 2004). Other factors also have a heparin-binding cluster of positively charged aminoacids, which, nevertheless, differs from the collage-like domain in its structure (Ori et al., 2008). For some proteins, such as C1q, for example, the consensus aminoacid sequence(s) of the heparin-binding site have been identified (Garlatti et al., 2010). Arginine and Lysine are the amino acids, forming positively charged clusters arranged as a positively charged groove (Doi et al., 1993). Arginine provides a more stable hydrogen bond and a stronger electrostatic interaction with sulfate groups than Lysine does. Additionally, in the structure of heparin-binding domains, neutral amino acids such as Serine and Glycine are very common.

Positively charged heparin-binding clusters of amino acids are also capable of high-affinity binding a variety of polyanions, primarily DNA molecules, low-density lipoproteins, lipopolysaccharides, and carbohydrates. For C1q, the sites responsible for binding heparin and dsDNA differ in their consensus sequence but are structurally enclosed in one another (Garlatti et al., 2010).

The well-known feature of both dsDNA and heparin is their negative charge of the molecule. It can be presumed that the positive charge of the cancer stem cell surface, which is formed by multiple positively charged domains located in proteins belonging to different functional groups, is a competitive substrate for both types of molecules. Heparin is known to bind to certain growth factor receptors, various proteoglycans, scavenger receptors, and caveolae-associated glycoproteins (Doi et al., 1993; Ori et al., 2008, 2011). It can be supposed that it is these types of molecules that determine the positive charge of the cell surface and play the key role in its interaction with extracellular dsDNA*.*

Thus, one of the possible elements of the cell surface, which is capable of binding extracellular dsDNA due to a molecular interaction, has been identified. This is the heparin/polyanion-binding site composed of either clusters of positively charged Arginine/Lysine within the collagen-like domain (Doi et al., 1993; Deprez and Inestrosa, 1995; Delacoux et al., 2000; Muñoz and Linhardt, 2004), clusters of positively charged amino acids having no homology with the collagen-like domain (Ori et al., 2008), or a consensus sequence of amino acids (Garlatti et al., 2010).

A generalized logic line of the results obtained using three independent approaches and provided in paragraph 4.2, namely, 1) direct assessment of the migration of TAMRA + cells to the cathode, 2) retaining the dsDNA probe-binding potency upon negating the negative charge by BB41, and, at the same time, 3) dose-dependent abrogation of the dsDNA probe-binding potency upon negating the positive charge of cells by negatively charged heparin, indicates the presence of a positive charge on cells capable of internalizing the TAMRA-dsDNA probe.

#### 3.2.4 Molecular characterization of the surface elements of TAMRA + cells, determining the interaction of cells with the TAMRA-dsDNA probe

The body of the data obtained indicated that some surface components present on positively charged cells participate in the process of dsDNA binding. As follows from the published data, these factors have heparin-binding domains in their structure, which determines the competitive character of heparin and dsDNA binding. Some of these factors, apparently, are not immobilized in the cytoplasmic membrane and, thus, are capable of free drifting along the cell surface being affected by the electric field. Such properties are known to be inherent for glycocalyx proteoglycans, scavenger receptors, PGI-AP, etc (see references in the “Discussion”) or, in other words, for the components of the glycocalyx and caveolae- and clathrin-dependent import structures. A series of experiments indicating that cell surface proteins are the factors of dsDNA binding by cancer stem cells has been conducted.

##### 3.2.4.1 Effect of EDTA and Ca++ ions on the interaction of cells with TAMRA-dsDNA

The outer pericellular space of a variety of cell types, including stem and cancer stem-like ones, is formed by the glycocalyx, which consists of membrane-associated receptors, proteoglycans, and proteins of the extracellular matrix. Many of these molecules are charged and associated with divalent metal ions. The effect of EDTA-mediated depletion of divalent metal ions, as well as following saturation with Ca++ ions, on the efficacy of TAMRA-dsDNA binding was estimated in Krebs-2 and EBV + B-lymphoma cells (Figures 4C–E). Divalent metals used in the reported concentrations exerted no effect on dsDNA binding to Krebs-2 and EBV + B-lymphoma cells.

##### 3.2.4.2 Effect of the exposure of Krebs-2 cells to trypsine, PrK and collagenase solely and in combination with heparin

Effects of cells’ exposure to trypsin, PrK, and collagenase on the percentage of cells efficiently binding the extracellular TAMRA-dsDNA probe have been assessed. The results obtained indicate that the cell surface components (glycocalyx and cholesterol microdomains) affected by these proteolytic enzymes play an active role in the process of dsDNA interaction with cells. The preexposure of cells to these enzymes results in the decreased content of TAMRA + cells (*p* < 0.01 for collagenase and trypsin and *p* < 0.05 for PrK) (Figure 4F), and collagenase affects this process reliably less than trypsin and PrK (*p* < 0.05 and *p* < 0.01 respectively). Otherwise, if cells are first incubated with TAMRA-dsDNA, the treatment with the proteases results in the minimal percentage of TAMRA + cells for all types of the enzymes used (*p* < 0.01) (Figure 4G). This most likely means that 1) this is exactly the number of cells that internalized the dsDNA probe (DNA fragments are in the internal compartments of cells) and 2) prior to the treatment with proteases, a significant amount of TAMRA-dsDNA is just bound to the glycocalyx/cholesterol microdomains component without internalization, providing a seeming increase in TAMRA + cells. Taken together, these data indicate that the cell surface components bind TAMRA-dsDNA without internalization, at least within the described time span, causing the seeming percentage of TAMRA + cells to be increased. The true internalization is either absent or in the initial stage. It can be presumed that cells bind DNA fragments using specific components of the glycocalyx/cholesterol microdomains and hold them for a long time, ensuring their high local concentration in the immediate vicinity of the cell membrane. This process is probably to be an essential step of the trafficking of extracellular dsDNA fragments into the cell.

At the second stage of this series of experiments, the combined effects of proteases and heparin on the DNA binding have been estimated. In the preliminary experiments, it was found that 30 min of exposure of cells to proteases did not affect the percentage of TAMRA + cells. With this regard, the time interval of 30 min was used as the basic protease treatment time. It turned out that preexposure of cells to proteases for 30 min abrogated the inhibitory effects of heparin (*p* < 0.05), which otherwise resulted in the decreased content of TAMRA + cells (Figure 4H).

Thus, it has been established that 1) the lack of divalent metal ions does not affect the percentage of TAMRA + cells, 2) the exposure of cells to proteolytic enzymes (trypsin, PrK, and collagenase) results in a significant decrease in the content of TAMRA + cells both prior to and after the incubation with TAMRA-dsDNA, and 3) the exposure of cells preexposed to proteolytic enzymes to heparin does not affect the percentage of TAMRA + cells.

##### 3.2.4.3 Effect of PrK on EBV + B-lymphoma cells

Experiments on treating cells with PrK and TAMRA-dsDNA were repeated in the EBV + B-lymphoma model with regard to the effect of such treatment on the mobility of treated cells in the free volume. PrK was used as the main proteolytic enzyme due to its most pronounced effect among those tested in experiments with Krebs-2 cells. In this series of experiments, the mobility of cells during their electrophoretic separation in a free volume was estimated. Cells treated with PrK were subjected to electrophoresis. After separation, both “+” and “–” fractions of cells were incubated with TAMRA-dsDNA (n = 4) (Table 1). The results obtained indicate that electrophoretic migration of cells toward the anode either does not depend at all or depends only partially on the cell surface protein components susceptible to PrK-mediated hydrolysis. TAMRA + cells of EBV + B-lymphoma seem to have the positively charged surface, which is determined by the protein fraction, the partial hydrolysis of which does not abrogate their migration to the negatively charged electrode. These data suggest that the positive charge is distributed throughout the entire volume of the cell surface, including both the part exposed toward the outer space and that adjacent to the cytoplasmic membrane itself.

The results obtained suggest the role of surface proteins in the process of interaction of cells with dsDNA. Moreover, the data obtained indicate that the seeming percentage of TAMRA + cells is determined by two subpopulations, one of which is the cells just holding the labeled dsDNA probe at their surface, and the other one is the cells actually internalizing the probe.

##### 3.2.4.4 The rate of TAMRA-dsDNA accumulation in the internal compartments of Krebs-2 cells pre- and post-treated with PrK

It was necessary to realize the obligatoriness of the protein coating at the surface of TAMRA + cells, susceptible to proteolytic enzymes, for the process of DNA internalization. As it was mentioned above, PrK exerted the most profound proteolytic effects (estimated by the decrease in the content of TAMRA + cells) and, thus, was chosen as the main enzyme for further experiments. The rate of the TAMRA-dsDNA probe uptake by individual cells both pre- and post-exposure to PrK has been estimated in the Krebs-2 model. These experiments were supposed to clarify the role of surface proteins in the process of dsDNA import into cells. Significant differences have been observed both in the rate of accumulation and in the fluorescence intensity of TAMRA-labeled material in cells before (*p* < 0.05) and after exposure to PrK. In the first case, there was observed a continuous accumulation of the labeled material with a maximal intensity of 250 ± 50 conventional units (CU) reached after 30 min of incubation. In the second case, the process of accumulation has been found to cease after 7 min of incubation and the final fluorescence intensity of cells was 37 ± 14 CU, which is equal to that of native cells after 1 min of incubation with TAMRA-dsDNA (Figure 4I). Intracellular localization of TAMRA-dsDNA has been confirmed by confocal microscopy (Figure 4J). This result presumed the essential role of some surface component, which is resistant to PrK under experimental conditions, in the process of binding and import of TAMRA-dsDNA. Other components of the cell surface determine only the binding of dsDNA but not the internalization.

The results of experiments on treating cells with proteases, followed by exposure to heparin, suggest the following possible localization of DNA-binding sites:

• on the cell surface, there are both proteins which contain both heparin- and DNA-binding domains and proteins which contain DNA-binding domains only, and the latter ones are less susceptible to proteolysis;
• on the proteins responsible for concurrent binding of both heparin and dsDNA, some of the DNA-binding domains do not overlap with heparin-binding ones and are presumably located in the immediate vicinity of the membrane and, thus, cannot be affected by proteolytic enzymes, remaining intact and mediating the process of dsDNA internalization detection after exposure to proteases and heparin; and
• scavenger receptors are known to be internalized as a part of caveolae and then re-exposed on the surface of the cytoplasmic membrane (Nichols et al., 2001); thus, it can be presumed that the internalized fraction of these receptors, which cannot be affected by heparin during the relatively short-term exposure, undergoes the re-exposition after heparin is being washed off and participates in the binding and internalization of dsDNA fragments.

To validate the hypothesis supposing the existence of several types of cells, such as 1) capable of tight binding the TAMRA-dsDNA fragments without internalization, 2) capable of internalizing these fragments, and 3) cells belonging to a variety of intermediate subtypes, there were conducted experiments on assessing the rate of the fluorescent material disappearance from the surface of cells treated with PrK after their incubation with TAMRA-dsDNA. In the case of Krebs-2, a vast majority of cells taken from the near-death stage ascites (14–17 days of the tumor development with an ascites volume of 10–20 ml) were shown to expose dsDNA-binding factors on their surface. For these cells, it was found that dsDNA-binding factors of the cell surface can have different capabilities of dsDNA probe internalization and can be located both on cells of different types and on the same cell. In other words, at the same time, there are two types of cells with two variants of the fate of bound dsDNA fragments: 1) to be retained exclusively at the cell surface without internalization and exfoliated during the exposure to PrK (Figure 4K1-1,2) and 2) to be gradually, within 2-h exposure to 50 μg/ml of PrK, transferred from the surface into the internal compartments of cells (Figure 4K1-3,K2). It can be presumed that in this case, we have a deal with two types of tumor cells: 1) TSCs (which usually account for ∼1%) and 2) committed Krebs-2 cells with the noted changes.

The data obtained confirm the presumption that there are two concurrent types of cells with two variants of interaction with dsDNA. The first type of cells binds the dsDNA probe and retains it on the surface without internalization. The second type binds the probe and gradually, during a certain time (∼2 h), internalizes the labeled material into the intracellular compartments.

#### 3.2.5 Determination of the mechanism of the dsDNA probe internalization into cells

##### 3.2.5.1 Effect of disruption of some endocytosis mechanisms on the content of TAMRA + cells in the models of Krebs-2 and EBV + B-lymphoma

The results provided in the previous sections indicate that TAMRA-labeled extracellular DNA fragments mark the TSCs. In this part of the investigation, an approach supposing the disruption of the known mechanisms of endocytosis with the subsequent estimation of their impact on the content of TAMRA + cells was used. Effects of nine inhibitors affecting different endocytosis mechanisms have been estimated in both cellular models used. Dynasore was used as an inhibitor of dynamin-dependent endocytosis, chlorpromazine was used as an inhibitor of clathrin-dependent endocytosis, nystatin and MβCD were used as inhibitors of endocytosis mediated by the lipid rafts, including the caveolin-dependent way, cytochalasin D and EIPA were used as inhibitors of macropinocytosis, wortmannin was used as an inhibitor of phosphatidylinositol 3′-kinases, and sodium azide was used as an inhibitor of ATP synthesis.

**FIGURE 5:**
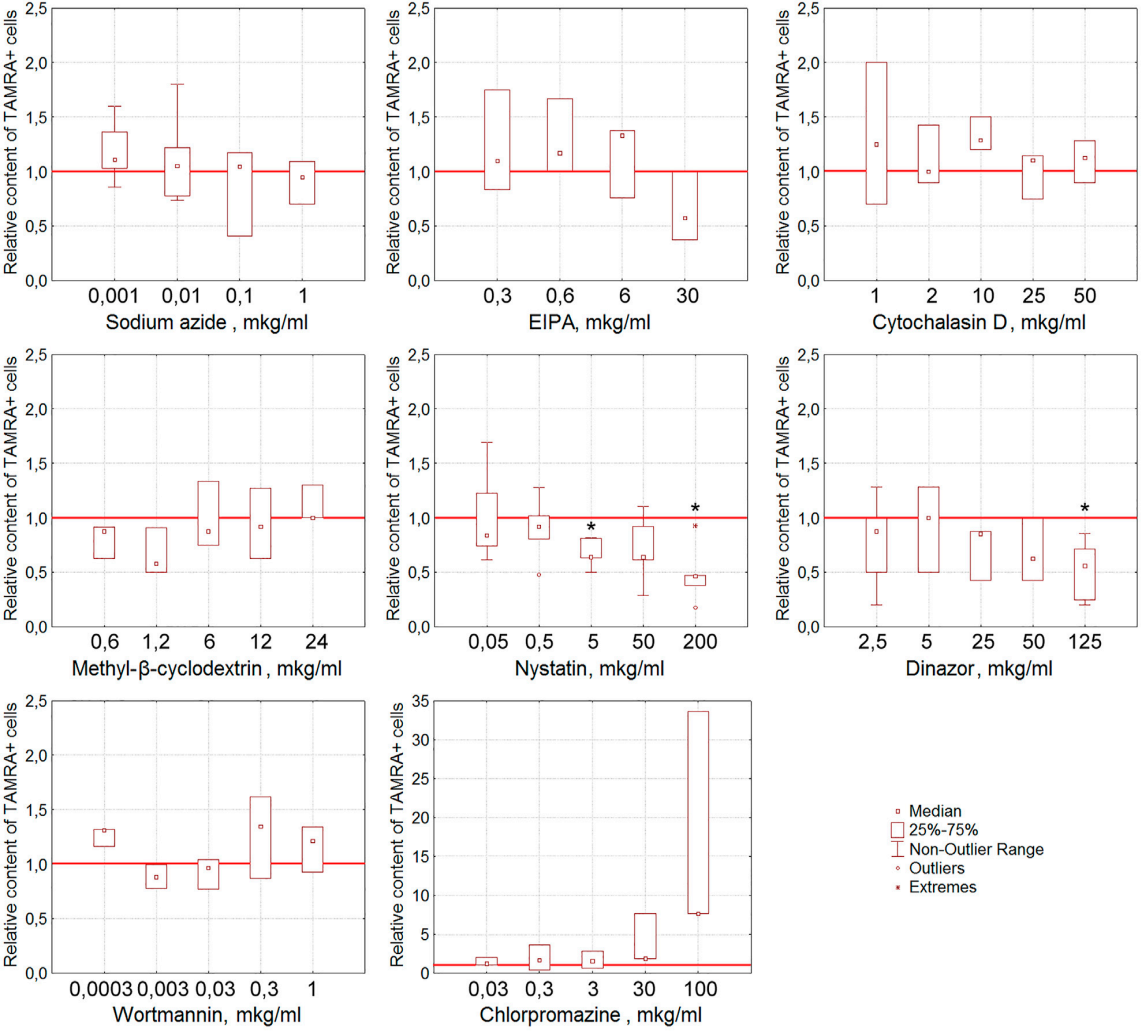
Effect of various compounds on the binding of TAMRA-dsDNA to Krebs-2 cells. The plots display the content of TAMRA + cells after their exposure to appropriate compounds at different concentrations (n = 3–6). Asterisks denote the values reliably differing from the unexposed control (*р*<0.05 according to the Kruskal–Wallis ANOVA test). The red line denotes the content of TAMRA + cells (taken as 1) in the bulk of Krebs-2 cells unexposed to inhibitory compounds.

Nystatin, the inhibitor of the caveolin-dependent way of internalization, has been shown to reduce the content of TAMRA + Krebs-2 cells in a statistically significant (*p* < 0.05), dose-dependent manner (Figure 5). Dynasore, the inhibitor of dynamin-dependent endocytosis, also caused the lessening of the TAMRA + cell percentage at the maximal concentration used (125 μg/ml, *p* < 0.05).

**FIGURE 6:**
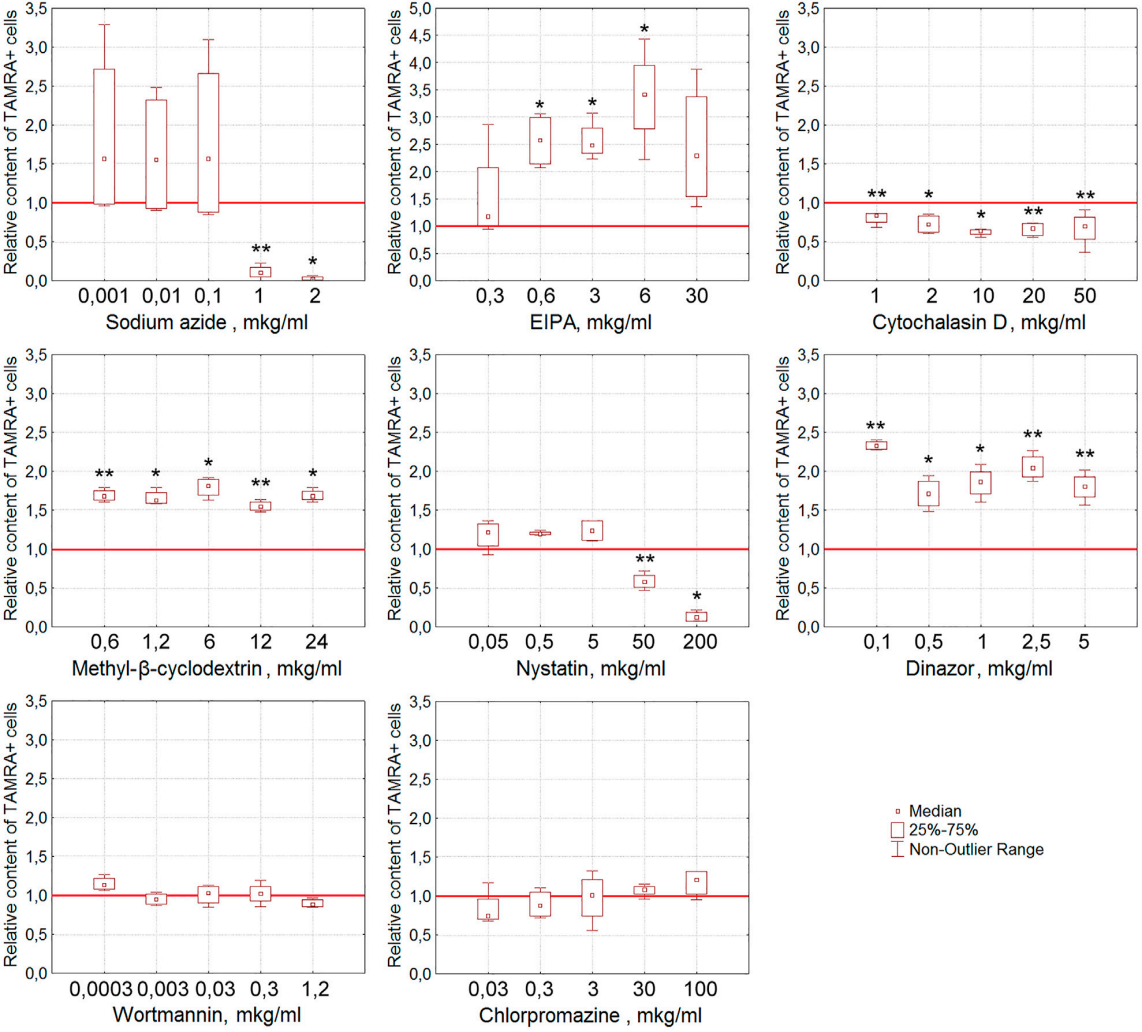
Effect of various compounds on the binding of TAMRA-dsDNA to EBV + B-lymphoma cells. The plots display the content of TAMRA + cells after their exposure to appropriate compounds at different concentrations (n = 4). Asterisks denote the values reliably differing from the unexposed control (*p<0.05, **p < 0.01 according to the Kruskal–Wallis ANOVA test). The red line denotes the content of TAMRA + cells (taken as 1) in the bulk of EBV + B-lymphoma cells unexposed to inhibitory compounds.

For EBV + B-lymphoma, the content of TAMRA + cells was also shown to reduce reliably after their exposure to nystatin (*p* < 0.01 and *p* < 0.05 for concentrations of 50 and 200 μg/ml, respectively) (Figure 6). Sodium azide almost completely abrogates the interaction of dsDNA with EBV + B-lymphoma cells (*p* < 0.01 and *p* < 0.05 for concentrations of 1 and 2 μg/ml, respectively) in a dose-dependent manner that testifies to the dependence of this process on ATP. Cytochalasin D, the inhibitor of actin polymerization, interferes with dsDNA binding to cells at all concentrations tested (*p* < 0.01 for concentrations of 0.1, 20, and 50 μg/ml and *p* < 0.05 for 2 and 10 μg/ml) in a statistically significant manner.

It was noted that many of the compounds used corrupt the integrity of the membrane of the exposed tumor cells, resulting in a significant increase in the percentage of TAMRA-fluorescent cells. Thus, the percentage of TAMRA + cells determined by the FACS assay increased in a statistically significant manner upon exposure to EIPA (*p* < 0.05 for concentrations of 0.6, 3, and 6 μg/ml), MβCD (*p* < 0.01 for concentrations of 0.6 and 12 μg/ml and *p* < 0.05 for 1.2, 6, and 24 μg/ml), and Dynasore (*p* < 0.01 for concentrations of 0.1, 2.5, and 5 μg/ml and *p* < 0.05 for 0.5 and 1 μg/ml). Cytological assays also indicated the corruption of the cytoplasmic membrane as the cause of non-specific binding of TAMRA-dsDNA to cells.

##### 3.2.5.2 Real-time PCR using DNA isolated from cells incubated with the TAMRA-dsDNA probe after their exposure to the appropriate inhibitory compound

At the initial stages of the study, it was presumed that the reduced percentage of TAMRA + cells would reflect the inhibition of the extracellular dsDNA probe internalization. The experiments performed, however, revealed that the interaction of cells with dsDNA does not always imply its internalization into the cell. This, in turn, could mean the incorrectness of conclusions regarding the inhibitory effects of the tested compounds based on the estimation of changes in the content of TAMRA + cells. To resolve the issue, experiments on assessing the inhibitory effects of the tested compounds on the dsDNA internalization process were repeated using the following modified approach. Cells preexposed to the appropriate compound at the average selected concentration (or at concentration of maximal effect) were incubated with the TAMRA-dsDNA probe. After this, cells were subjected to the excessive treatment with DNase and PrK, followed by the total DNA isolation. The isolated DNA was further used as a template in RT-PCR with primers specific to the TAMRA-dsDNA probe, as well as to the appropriate reference genes, actin in the case of Krebs-2 and DNA-polymerase in the case of EBV + B-lymphoma. The results are shown in Tables 2 and 3.

**TABLE 2:** Relative content of internalized TAMRA-dsDNA in the internal Krebs-2 cell compartments after their exposure to different inhibitors.

Inhibitor	# of experiment			Median
	1	2	3	
Heparin	0.93	1.00		0.97
Sodium azide	1.83	1.77		1.80
EIPA	2.99	1.93		2.46
Cytochalasin D	0.19	0.27		0.23
MβCD	21.63	21.63	5.26	21.63^a^
Nystatin	0.45	0.30	0.42	0.42^a^
Dynasore	2.12	0.80	0.51	0.80
Wortmannin		4.50	7.19	5.85
Chlorpromazine	33.85	23.38		28.62

**Comment:** Relative values, calculated as the ratio of the amount of TAMRA-dsDNA, internalized by cells exposed to certain inhibitors (normalized to the amount of native murine DNA, in each sample) to that in intact control cells, are shown. Values below 1 indicate the suppression of the internalization process in treated cells relative to intact ones.

a reliable differences relative to the control, *р*<0.05 according to the Mann-Whitney *U*-test.

**TABLE 3:** Relative content of internalized TAMRA-dsDNA in the intracellular compartments of EBV + B-lymphoma cells after their exposure to different inhibitors.

Inhibitor	# of experiment			Median
	1	2	3	
Heparin	25.82		1	13.41
Sodium azide	0.83		1.47	1.15
EIPA			1.84	1.84
Cytochalasin D	0.11		0.14	0.13
MβCD	0.17		1.32	0.75
Nystatin	3.77	3.53		3.65
Dynasore		5.73		5.73
Wortmannin		0.20	0.23	0.22
Chlorpromazine	0.16	0.76		0.46

**Comment:** Relative values, calculated as the ratio of the amount of TAMRA-dsDNA, internalized by cells exposed to certain inhibitors (normalized to the amount of native human DNA, in each sample) to that in intact control cells, are shown. Values below 1 indicate the suppression of the internalization process in treated cells relative to intact ones.

The results obtained were compared to those of the initial experiments based on simple assessing of the reduction of TAMRA + cell content after exposure to the appropriate compound. The results turned out to be more or less the same in both cases.

In the case of Krebs-2, two independent approaches revealed the dsDNA internalization process inhibition by dynasore and nystatin, which affect the endocytosis. In experiments with real-time PCR, cytochalasin D was found to suppress the internalization also. After taking all the discovered peculiarities of the dsDNA internalization into account, it was concluded that cytochalasin D exerts the most profound effect on this process as compared to dynasore and nystatin. Thus, it can be suggested that in the model of Krebs-2 cells, the process of dsDNA import utilizes endocytosis (abrogated by dynasore and nystatin) and macropinocytosis (abrogated by cytochalasin D) (Table 2). The results of early experiments indicating the corruption of the cellular membrane due to exposure to some compounds used that resulted in aberrantly increased “internalization” of TAMRA-dsDNA fragments were also confirmed.

Sodium azide exerted almost no effect on dsDNA internalization, indicating this process to be ATP-independent.

As it was noted above, heparin significantly decreases the percentage of TAMRA + cells. PCR assay conducted with Krebs-2 cells revealed that such a decrease is due to the abrogation of superficial dsDNA binding only, while the true internalization is unaffected by heparin, since the detected content of the dsDNA probe in cells exposed to heparin was equal to that in unexposed ones. This, in turn, means that high pericellular concentration of dsDNA is most probably not mandatory, neither for the act of internalization itself nor for increasing its efficacy.

In the same experiments, the absolute content of the TAMRA-dsDNA probe in the internal Krebs-2 cell compartments under regular conditions has been assessed. It turned out that a single cell contains ∼500 copies of the TAMRA-labeled double-stranded *Alu* fragment of 500 bp size, or 250,000 bp total, which constitutes 0.008% (∼0.01%) of the normal haploid genome (∼3 × 10^9^).

Similarly, in the case of EBV + B-lymphoma, results of real-time PCR do not generally contradict those of experiments on assessing the percentage of TAMRA + cells after exposure to inhibitors (Table 3). The reliable inhibition has been observed for cytochalasin D, the inhibitor of macropinocytosis, for MβCD, the inhibitor of caveolar and flotillin-dependent endocytosis due to the disruption of lipid rafts, for wortmannin, the blocker of receptor-mediated endocytosis, and for chlorpromazine, the inhibitor of clathrin-dependent endocytosis. In other words, internalization is affected by the compounds, which impact the factors of both clathrin-dependent caveolar endocytosis and macropinocytosis. Sodium azide was noted to have no effect on the amount of dsDNA internalized, although in the first series of experiments, almost complete abrogation of DNA binding to cells after their exposure to this compound has been observed. This implies that the process of internalization itself does not depend on the presence of ATP molecules, while the process of DNA binding to the factors of the cell membrane essentially does. Similarly, EIPA exerts almost no effect on the internalization. For heparin, results turned out to be contradictory. There were increased amounts of the dsDNA probe inside the cells, which we believe were associated with their long-term incubation in the culture medium, which results in changing the protein composition of the cell membrane, aberrant effect of heparin, and corruption of the cell membrane integrity.

The results obtained presume the involvement of caveolin-dependent transport and macropinocytosis in the process of dsDNA import into Krebs-2 cells. In the case of EBV + B-lymphoma, this process involves caveolar and clathrin-dependent mechanisms, as well as macropinocytosis.

Effects of the used inhibitors on dsDNA binding and internalization have been collated (Table 4).

**TABLE 4:** Collation of the impact of inhibitors on dsDNA binding and internalization.

Cellular model	Abrogation of the binding to cells (% of TAMRA + cells)	Abrogation of the internalization
Krebs-2		Nystatin
	Nystatin	Dynasore
	Dynasore	Cytochalasin D
EBV+ В-lymphoma		Cytochalasin D
	Cytochalasin D	MβCD
	Nystatin	Wortmannin
	Sodium azide	Chlorpromazine

In Krebs-2 cells, nystatin and dynasore turned out to affect both the binding and internalization of the dsDNA probe, while cytochalasin D has no effect on the binding but abrogates the internalization.

In the case of EBV + B-lymphoma, the internalization was inhibited by MβCD, wortmannin, and chlorpromazine, which, however, had no effect on the binding of dsDNA to the cell membrane. Cytochalasin D was found to abrogate both the processes. Sodium azide drastically decreased the binding, but not internalization, presuming the role of ATP in the process of chemical/molecular interaction of dsDNA with the surface factors of EBV + B-lymphoma cells.

Additionally, experiments were conducted on exposure of Krebs-2 cells to 500 kDa dextran sulfate, which is known to block the ability of scavenger receptors to bind ligands, primarily heparin and low-density lipoproteins (Basu et al., 1979; Tsubamotoa et al., 1994; Nishinaka et al., 2020). Efficacy of the internalization was assessed using real-time PCR after the treatment of cells with PrK and DNase. It turned out that cells exposed to dextran sulfate completely lose their capability of both binding and internalizing the dsDNA probe. According to the results of real-time PCR, the relative content of internalized dsDNA was 0.08, which is absolutely consistent with the results of microscopic examination that revealed no TAMRA + cells (data not shown). This implies the role of scavenger receptors in the process of dsDNA internalization by TSCs.

## 4 Discussion

As was mentioned earlier, the formation of spheres in the culture of EBV + B-lymphoma cells occurs as a quick (∼20 min) aggregation of cells around the sphere-initiating center, which mandatorily contains at least one TAMRA + cell and looks like an electrostatic attraction between TAMRA + cell and its TAMRA– counterparts. Moreover, a thorough examination of this process revealed no signs of active cell movement, such as pseudopodia formation and others. This allowed us to presume that these two types of cells bear different charges and, thus, electrostatically attract each other.

To verify this observation, the model of Krebs-2 ascites carcinoma, which has been investigated in our laboratory for a very long time, was used additionally. For these cells, it was experimentally established that TAMRA + cells are tumorigenic stem-like ones (Dolgova et al., 2014). For both models, Krebs-2 and EBV + B-lymphoma (data in preparation), RNA-sequencing procedure was conducted, which demonstrated that TAMRA + cells overexpress the characteristic “stemness” genes and, at the same time, have the HSC-specific marker CD34 (for Krebs-2) and CD133 (for EBV + B-lymphoma) on their surface (Dolgova et al., 2013; Potter et al., 2016, 2017). Some details of the extracellular dsDNA internalization process have been clarified for this type of cell. Thus, it was established that extracellular dsDNA, after internalization, can be detected both in the cytoplasm and in the nucleus, and its distribution among the main cell compartments is irregular and depends on the type of DNA (linear dsDNA fragments, supercoiled plasmid DNA, etc.) (Dolgova et al., 2016a; Potter et al., 2018).

To determine the total charge of TAMRA + cells in both cell models, two series of experiments have been carried out. Cells were electrophoretically separated in a free volume, and “+” and “–” fractions were collected and incubated with TAMRA-dsDNA. “–” fraction was found to be significantly enriched with the cells capable of internalizing the TAMRA-dsDNA probe. In the other series of experiments, cells were exposed to cationic dye Basic Blue GRL 41 and TAMRA-dsDNA in different orders. TAMRA + cells were proved to remain unstained with this dye, that is, have a non-negative surface charge.

The complex of data obtained suggested with a high degree of probability that one of the main differences of Krebs-2 TAMRA + stem-like cells from their TAMRA- counterparts is the positive surface charge, which determines the initial association of extracellular dsDNA with the cell surface.

There were additionally conducted experiments on assessing a competitive interaction with heparin, which, similarly to dsDNA, has a strong negative charge. It was shown that heparin competitively inhibits the process of dsDNA internalization.

For the thorough cytological examination of the interaction of TAMRA-dsDNA with the cell surface, a method of micro-gel-electrophoresis has been developed. Using this approach, it was found that

• the labeled dsDNA material detected inside the cell retained its spatial location during micro-gel-electrophoresis;
• the labeled dsDNA material “drifted” across the membrane toward the anode; and
• the labeled dsDNA material exfoliated completely from the cell surface during micro-gel-electrophoresis.

The last two observations, namely, “drifting” and exfoliation of TAMRA-dsDNA, turned our understanding of the internalization process upside down. It became clear that chemical/molecular interaction with the specific surface factors is the second step in the binding of dsDNA to cells.

Thus, the general pattern of the initial steps of the interaction between polyanions and the cell surface could be proposed as follows. The charge is the factor, which initiates the interaction of dsDNA with the cell surface, and the observed exfoliation testifies to this. But the charge does not determine the strong retention of dsDNA fragments in the direct vicinity to the cell membrane and their internalization into the cell.

After the initial electrostatic attraction mediated by the charge, dsDNA is “fixed” on the cell surface by the strong interaction with the cell surface proteins and, being exposed to the electric field, drifts across the cell membrane toward the anode without dissociation from it. Such a drifting is presumed to occur due to 1) the negative charge of the dsDNA molecule and 2) strong membrane anchoring of the proteins dsDNA interacts with. Finally, heparin competes with dsDNA for the binding to the cell surface but does not seem to abrogate its internalization, despite the fact that heparin is also capable of being internalized (see references above). It may mean that there are the particular factors determining the internalization of dsDNA, exactly. As a result of the study conducted, we have formulated two fundamental, in our opinion, questions.

• How is the positive charge of TSCs formed (or what does it depend on)?

Analysis of the published data revealed that within the generally accepted paradigm, all eukaryotic cells have the negatively charged surface (Le et al., 2019). It is also believed that all tumor cells similarly have the negatively charged surface. Thus, for example, in some studies using positively charged nanoparticles for the selection of cells by their charge, it was demonstrated that tumor cells from 22 specimens were charged negatively (Chen et al., 2016; Le et al., 2019). Nevertheless, in the cited article, it was also demonstrated that non-tumor cells have either neutral or marginally positive charge. The negative surface charge is presumed to correlate with glycolysis and excessive excretion of lactic acid (Forest and Pourchez, 2017; Nandhakumar et al., 2017). It was the only article we found discussing the possibility of electrostatic interactions between stem cells and stromal cells of the stem niche due to different surface charges of these cells (Fuchs and Blau, 2020b). Thus, the facts presented testify to the possibility of the existence of positively charged cells in principle.

Results reported in the current study differ from the commonly accepted concept and presume that TSCs, in contrast to their committed progeny, have the positive surface charge. We could not find any experimental reports supporting our findings and, therefore, could not rely on the experimental background discussing the results obtained, and thus, we propose our own original concept.

• What cell surface factors determine the binding and internalization of the dsDNA probe?

In the investigation conducted, it was established that three particular polyanions, dsDNA, heparin, and dextran sulfate, competitively interact with the surface proteins of TAMRA + TSCs of Krebs-2 carcinoma and B-lymphoma. Confocal microscopy and experiments on competitive interplay of dsDNA, heparin, and dextran sulfate with TAMRA + TSCs, which specifically bind polyanions, testify to the existence of the well-developed glycocalyx on these cells. Glycocalyx is the gel-like shell found on the surface of cells throughout the human organism and is a physical barrier between the cell and its surrounding microenvironment (Tarbell and Cancel, 2016). The main components which form the structure of glycocalyx are proteoglycans, glycosylated core glycoproteins, and proteins of the extracellular matrix. In the structure of glycocalyx, there are also various membrane-anchored proteins, associated with lipid rafts or with clathrin complexes. Glycocalyx is charged negatively. The negative charge depends on the glycosaminoglycan side chain sulfation. The charged glycocalyx network is believed to act as a macromolecular strainer, which repels negatively charged molecules, as well as erythrocytes and platelets. Some amphoteric molecules with a minor positive charge, such as albumin, are also capable of binding to glycocalyx components (Pries et al., 2000; Reitsma et al., 2007; Becker et al., 2010).

Many of the glycocalyx components are known to bind negatively charged heparin and dextran sulfate. The main determinants of such a binding are clusters of positively charged amino acids and collagen-like domains, ubiquitously presented in the glycocalyx components belonging to functionally and structurally different groups of proteins. Heparin-binding sites are featured with the presence of clusters of positively charged aminoacids, which form ion pairs with negatively charged sulfate of the polysaccharide. Non-electrostatic interactions, such as hydrogen bonds and hydrophobic interactions, can also contribute to the stability of heparin–protein complexes (Muñoz and Linhardt, 2004; Ori et al., 2008).

Glycocalyx and forming its proteoglycans/glycoproteins are characteristic of tumor/TSCs. To date, there are about 30 tumor stem-like cell surface glycoproteins that have been identified. The factors of glycocalyx participate in the totality of processes ensuring the functionality of TSCs. Glycocalyx plays an essential role in the receptor–ligand interaction in tumor cells and their environment, ensuring the epithelial-to-mesenchymal transition, exit to circulation, anoikis resistance, extravaztion, and survival in the “alien” environment. Proteoglycans affect the cell growth and proliferation through the interaction with growth factors. Overexpression of some glycoproteins, for example, Mucin 1, correlates with the development of aggressive cancers (Knelson et al., 2014; Mitchell and King, 2014; Paszek et al., 2014; Mallard and Tiralongo, 2017; Barnes et al., 2018; Kuo et al., 2018; Vitale et al., 2019; Ahrens et al., 2020). Thus, glycocalyx is commonly accepted as a structural feature of TSCs, which ensures a variety of their functions.

Experiments conducted within the frames of this study indicate that competitive effects of heparin (12–16 kDa) and dextran sulfate (500 kDa) in their relation to dsDNA (150 kDa) are different. Heparin competitively inhibits only the binding, while dextran sulfate inhibits both the binding and internalization.

Heparin is known to specifically bind to the so-called collagen-like domain, which is characterized either by a particular positively charged binding site or by a set of subsequent clusters of positively charged amino acids (Doi et al., 1993; Deprez and Inestrosa, 1995; Delacoux et al., 2000; Muñoz and Linhardt, 2004; Ori et al., 2008). Collagen-like domains are found in the structure of proteoglycans of the glycocalyx and class A scavenger receptors (Muñoz and Linhardt, 2004; Ori et al., 2008, 2011; Chiodelli et al., 2015; Meneghetti et al., 2015). Moreover, heparin binds to the particular site with the consensus amino acid sequence specific to the collagen-like domain of C1q factor of the complement (Garlatti et al., 2010). Finally, heparin can be bound and internalized by the class H scavenger receptors (Urano et al., 1997; Harris et al., 2008, 2009).

The data obtained indicate that dsDNA and heparin compete for the same binding domains, but this competition does not affect the internalization, implying the role of some other surface factor, which mediates the import of dsDNA into the cell.

**FIGURE 7:**
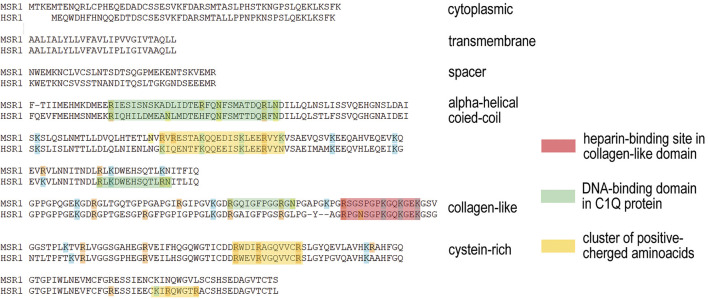
Amino acid sequence of collagen-like domain of class A scavenger receptors (Doi et al., 1993) with the site of binding acetylated low-density lipoproteins (highlighted in red). Analysis conducted indicates the presence of additional clusters of positively charged arginine and lysine (highlighted in yellow) and the consensus sequence of dsDNA-binding site (Garlatti et al., 2010) (highlighted in green). Such a combination of the highlighted sites capable of binding a variety of polyanions presumes the possible role for this class of scavenger receptors in the process of dsDNA internalization.

Scavenger receptors determine the internalization of a variety of ligands (PrabhuDas et al., 2017; Alquraini and El Khoury, 2020; Weigel, 2020). Class A of these receptors has a collagen-like domain, which forms a positively charged groove (Doi et al., 1993), and binds and imports polyanions, primarily acetylated low-density lipoproteins, into the cell. Analysis of the amino acid sequence of collagen-like domain of class A scavenger receptors indicates that besides the particular “+” groove formed by the cluster of positively charged amino acids, there are additional clusters of R and K amino acids. Moreover, in the structure of the domain in question, there are several crystallographically confirmed sites capable of specific binding dsDNA (Garlatti et al., 2010) (Figure 7).

These facts presume the possible role for this scavenger receptor in the binding and internalization of dsDNA. Thus, the general picture of the heparin and dsDNA interplay is as follows. Binding of dsDNA to the specific sites in the glycocalyx proteoglycans is to be competitively inhibited by heparin, as it is confirmed in the appropriate experiments. At the same time, dsDNA fragments are bound and imported by the scavenger receptors of presumably class A, while heparin is bound to the class H scavenger receptors. In this case, there is no competition between heparin and dsDNA, and pre-exposure to heparin does not affect the amount of internalized dsDNA probe, as it is confirmed by real-time PCR.

This conclusion is supported by the experiments on competitive inhibition of both the binding and internalization of dsDNA using 500 kDa dextran sulfate. These experiments indicate that dextran sulfate completely abrogates the binding of dsDNA both to the cell surface (data not shown) and to the receptors mediating its internalization (results of real-time PCR).

Dextran sulfate is an artificial polyanion, which competes with heparin for binding to class H scavenger receptors (Harris and Weigel, 2008; Nishinaka et al., 2020), inhibits class A scavenger receptor-mediated internalization of acetyl low-density lipoproteins and advanced glycation end products (Goldstein et al., 1979; Nishinaka et al., 2020), and can be internalized in complex with low-density lipoproteins through another type of scavenger receptor (Basu et al., 1979). Dextran sulfate functions as an “inducer of internalization” due to formation of an intermolecular bridge between extracellular domains. Dextran sulfate is known to be capable of simultaneously binding the extracellular domain of NRP1 and SREC-I scavenger receptor, which facilitates their coordinated internalization into endothelial cells (Narazaki et al., 2008). Dextran sulfate also binds the virion of *Herpes simplex virus* and the particular cellular receptor (in fact, a scavenger receptor) mediating the internalization of the pathogen (Dyer et al., 1997). Thus, dextran sulfate is a universal ligand for the surface molecules, including proteoglycans and scavenger receptors, which realize a variety of functions and are responsible for polyanion (heparin and dsDNA) binding, and this determines the competitive capabilities of dextran toward dsDNA.

### 4.1 Putative mechanism of the dsDNA probe endocytosis

Using the compounds inhibiting different endocytosis pathways, it was established that dsDNA internalization is realized through a caveolae-dependent mechanism in Krebs-2 stem-like cells, while in B-lymphoma ones, it involves both caveolae- and clathrin-dependent pathways.

There are three types of the receptor-mediated endocytosis known. These are clathrin-dependent, caveolae-dependent, and flotillin-dependent endocytosis. Clathrin-dependent endocytosis occurs through the formation of cytoplasmic membrane invaginations covered with clathrin molecules at the intracellular surface. Formed vesicles contain G-protein–coupled receptors, which mediate the transduction of signals activating a variety of intracellular pathways. Internalization of the vesicles is mediated by the clathrin clustering and polymerization of cavin (Hayer et al., 2010; Kaksonen and Roux, 2018; Mettlen et al., 2018).

Caveolae and caveolae-dependent endocytosis are associated with the formation of cholesterol microdomains, also known as cholesterol rafts. These structures also contain a variety of receptors grouped together, primarily scavenger receptors and glycosylphosphoinositol-associated proteins (GPI-APs). A distinctive feature of these formations is an anchoring of proteins in the raft, while caveolin clustering and cavin polymerization mediate the internalization of caveolae (Martinez-Outschoorn et al., 2015; Busija et al., 2017; Gu et al., 2017; Hubert et al., 2020).

There is another possible variant of dsDNA internalization through the flotillin-dependent endocytosis. Proteins of SPFH family (Stomatins, Prohibitins, Flotillins, and HflK/C) determine the formation of “signalosomes,” which are internalized through the endocytosis independent on clathrin/caveolin/dynamin. This type of internalization, similarly to the caveolae-dependent one, is associated with cholesterol raft-based microdomains (Otto and Nichols, 2011; Meister and Tikkanen, 2014). The internalization of flotillin microdomains with raft-anchored receptors is shown to be associated with a change in the membrane curvature (macropinocytosis) (Glebov et al., 2006; Frick et al., 2007; Bodin et al., 2014; Rose et al., 2014; Meister et al., 2017; Ferreira et al., 2020).

In the microdomains/rafts, there are grouped receptors of different functions. Some receptors belong to the group of scavenger receptors (PrabhuDas et al., 2017; Alquraini and El Khoury, 2020). Another important component of rafts is GPI-APs. Receptors of this type have no transmembrane domains and are anchored in the membrane through inositol immersed in the cholesterol-enriched islets (Zurzolo and Simons, 2016).

GPI-APs are involved in a variety of cellular processes and induce the phosphorylation of intracellular factors mediating the signal transduction, which results in proliferation, cytokine production, and oxidative “storm” (Martinez-Outschoorn et al., 2015; Hussein et al., 2020). Some GPI-AP receptors, for example, FGFR, have a heparin-binding site(s), which is also a putative dsDNA-binding one, indicating their possible involvement in dsDNA internalization (Fannon et al., 2000; Ori et al., 2008; Kinoshita, 2020).

Both types of cells can also internalize the dsDNA probe through the macropinocytosis. For the macropinocytosis, the participation of a branched actin network along the plasma membrane, which regulates the formation of protrusions at the dorsal surface of cells, in the uptake of extracellular components is characteristic. During the macropinocytosis, there is a lipid mediator, phosphatidylinositol-4,5-bisphosphate, produced in the area of protrusions, which is essential for binding the material to be uptaken (Canton, 2018).

Thus, caveolin, clathrin, and flotillin determine the three types of plasma membrane regions, participating in the process of internalization in general and being putative sites of dsDNA binding and internalization in particular. Proteoglycans/glycoproteins, scavenger receptors, and GPI-APs associated with these membrane regions are both the acceptors for the dsDNA fragments and factors of their import into the cell. The concentration of phosphatidylinositol-4,5-bisphosphate molecules in membrane foldings formed by the actin-mediated protrusions can also be a molecular marker of the specific membrane sites participating in the extracellular material uptake.

In the context of the reported study, any of the possible dsDNA internalization mechanisms, one of which can be the formation of a molecular bridge between the DNA acceptor and a scavenger receptor, can be considered. In this case, a high local concentration of the probe in the immediate vicinity of the cell surface due to association with non-internalizing proteins can be essential to provide a continuous dsDNA influx, regardless of its concentration in the extracellular environment.

The body of the results obtained indicates that the process of dsDNA binding occurs with the participation of proteins located on the surface of positively charged cells. These proteins have heparin- and dsDNA-binding sites, which are presumed to form the positive charge of the cell surface and can topologically be overlapped or located distantly from each other. DsDNA-binding sites are located in such a way that they retain their capability of binding ligands even after excessive treatment with PrK and heparin. The results indicate that the import of dsDNA fragments may occur with the participation of scavenger receptors. It is possible that the turnover of these receptors could be the cause of the lack of heparin-mediated inhibition of the internalization in experiments on heparin and PrK co-treatment (Nichols et al., 2001). Some dsDNA-binding proteins appear to have no strict localization on the membrane and drift across the cell surface being exposed to an electric field. Bound dsDNA fragments can be internalized into the cell by means of the caveolar pathway, which depends on lipid rafts, clathrin-dependent endocytosis, or macropinocytosis.

## 5 Conclusion

• The first factor determining the interaction of extracellular dsDNA with TSCs is their positive general charge. For dsDNA, the first stage of its interaction with the TSC is a Coulomb attraction of negatively charged dsDNA molecules to the positively charged surface of cells, which is common both for the *ex vivo* model of EBV + B-lymphoma and for the *in vivo* model of Krebs-2.
• The second phase of the interaction of extracellular dsDNA with TSCs is associated with the formation of a chemical/molecular bond of dsDNA fragments with the cell surface proteins.
• A specific dsDNA-binding site is present in the surface proteins both capable and incapable of being internalized into the cell. Both types of proteins can be presented both on the same cell and independently on different ones. In other words, there are several different types of surface proteins capable of binding dsDNA, which perform different functions associated either with the surface binding or with the import into the cell.
• Heparin and dsDNA compete for the sites of binding in surface proteins. The cell surface proteins interacting with dsDNA fragments have a heparin-binding domain, which contains either clusters of Lysine/Arginine, which form a positively charged groove, Lysine/Arginine clusters of no apparent homology to the collagen-like domain, or a domain containing particular overlapping sites binding heparin and dsDNA (C1q-like domain).
• Dextran sulfate completely abrogates the binding of dsDNA to the cell surface proteins. It also blocks the internalization of dsDNA fragments into Krebs-2 cells, suggesting a role of scavenger receptors in this process.
• In Krebs-2, dsDNA fragments are internalized *via* caveolae-dependent endocytosis and macropinocytosis. In the case of B-lymphoma, the process involves both caveolae- and clathrin-dependent endocytosis, as well as macropinocytosis. The data obtained presume that protein factors capable of binding dsDNA are spatially restricted by lipid rafts in the case of caveolar internalization, associated with particular receptors of the clathrin-dependent pathway, or located within membrane foldings of pinosomes formed.

Thus, the data characterizing the molecular picture of factors participating in the internalization of dsDNA have been obtained. We provide the concept scheme for the sequential experimental validation of events having a place during the interaction with extracellular DNA in Supplementary Material S3.

### 5.1 Final remark

Interaction of the TAMRA-dsDNA probe with TSCs of Krebs-2 carcinoma or B-lymphoma, which allows these cells to be distinguished by their increased fluorescence from the bulk of their unstained committed counterparts, is the novel property of poorly differentiated tumor cells, which can be utilized for their detection.

### 5.2 Further perspectives

As a result of the study conducted, the experimental design and logic for the further search for the factors of TSCs responsible for binding and internalizing dsDNA have been determined. In the presented report, it was established that TSCs of Krebs-2 carcinoma and EBV + B-lymphoma realize two types of interaction between dsDNA and a cell. In the first case, dsDNA binds to the surface proteins without the consequent internalization. In the second case, being bound, dsDNA undergoes the consequent internalization within caveolae or clathrin vesicles. It is proposed to identify the cell surface proteins, binding dsDNA fragments (as a competitor to heparin) and being candidates for factors, which mediate the “pure” dsDNA binding without the consequent internalization. It is also proposed to identify the surface proteins associated either with lipid rafts of caveolae or with receptors of clathrin vesicles, which mediate the internalization of dsDNA fragments (as a competitor to heparin and dextran sulfate). These proteins are presumed to be the candidates for the factors mediating the dsDNA probe internalization. In the second part of the study, the attempt to identify the aforesaid factors has been performed.

Finally, what is the “biological sense” of this, most likely, universal property of stem cells of various origins, including TSCs? In our new study (data in preparation), it was shown that after being internalized into HSCs, dsDNA induces the terminal differentiation, predominantly toward the granulocyte-macrophage lineage and, at the same time, activates the proliferation of progenitors and colony formation. It is known that trauma, ischemia, and inflammation result in releasing a large amount of self-DNA into the blood. Probably, upon reaching a certain concentration in plasma and/or interstitial fluids, this DNA stimulates resting HSCs, which triggers the proliferation of immune cells essential for wound healing and other reparative processes. Thus, one of the consequences of dsDNA internalization into HSCs is the transmission of a signal about the damage acquired. In the case of tumor cells, appearance of dsDNA molecules within, for instance, a dormant metastasis, which is a TSC migrated from the parent tumor node, can also be a signal for its proliferation and consequent growth of a distant tumor. It could also be a way to obtain nucleic acids as a nutrient. We also hypothesize the transit persistence of dsDNA molecules in HSCs (Dolgova et al., 2013; 2016a) to be a mechanism for continuous control of genetic status of cells across the whole organism, when HSCs can respond with reparative recombination with “attracted-its-attention” dsDNA fragments of altered nucleotide sequence or having some epigenetic modification(s) (what is the mechanism and what is the molecular basis for the implementation of such an “observational mode,” we do not know). Similar considerations are valid for TSCs also.

To comprehend this newly described biological phenomenon, which characterizes stem cells of various origins, completely, a brain-storm with a lot of experimental work is required.

## Data Availability

The original contributions presented in the study are included in the article/Supplementary Materials; further inquiries can be directed to the corresponding author.
