# Supplementary material for: The new general biological property of stem-like tumor cells Part I. Peculiarities of the process of the double-stranded DNA fragments internalization into stem-like tumor cells
Source: Front Genet. 2022 Sep 8;13:954395. doi: 10.3389/fgene.2022.954395 (PMC9492886; doi:10.3389/fgene.2022.954395)
Supplement: Supplementary file 1 [file DataSheet2.PDF]

Supplementary Material 3

The main point. TAMRA+ cells of Krebs-2 and Epstein-Barr Virus-induced (EBV+) B-lymphoma are tumor stem-like cells (TSCs) (Dolgova et al., 2014, 2016b, 2019; Potter et al., 2016, 2017). The main participants in the analyzed events are the TAMRA-labeled double-stranded DNA (dsDNA) probe and Krebs-2 and EBV+ B-lymphoma TSCs, which interact with the probe (detection system).

Sequential logic of the experiment is shown in the figure and is provided with the necessary comments.

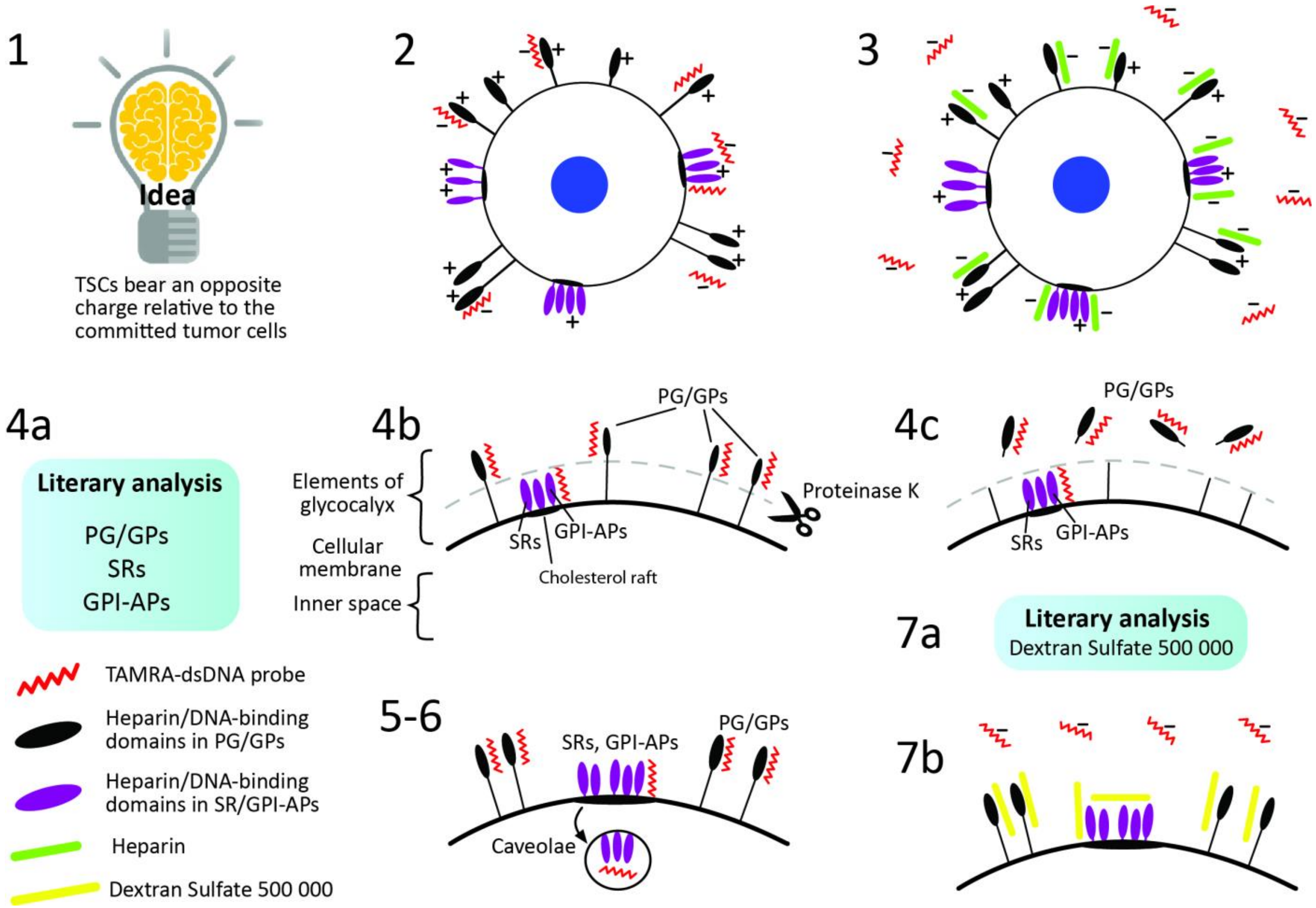

Figure S3-1. Schematic showing logic of the experimental procedures and how the sequence of evidence lead to the conclusions made in the investigation. A detailed description of each item is given below in the text.

1. The onset of the presumption that TSCs bear an opposite charge relative to the committed tumor cells. It is based on the analysis of the behavior of EBV+ B-lymphoma TAMRA+ cells during the initial stage of spheres formation, when the sphere-initiating center composed of ~9 cells is being formed as a result of the attraction and contact fixation between TAMRA+ cell and TAMRA- cells.

2. Proving the fact that TSCs bear an opposite charge relative to the committed tumor cells. Developing the experimental design and working out the systems of electrophoresis in a free volume and micro-gel-electrophoresis. Determining the fact that cells capable of binding TAMRA-dsDNA (as detected after the electrophoresis completed) predominantly migrate to the negative pole of the electrophoretic chamber. Estimation of the effect of negating the "negative" cell surface charge by the positively charged dye on the ability of these cells to bind the TAMRA-dsDNA probe revealed either complete absence of such an effect, or its very limited character. **Ascertaining the fact of general positive charge of TAMRA+ cells.**

3. Determining the carrier of the "positive" charge on the TSCs surface. Upon finding that the TAMRA-dsDNA probe interacts with positively charged cells, we have recalled our results published earlier (Dolgova et al., 2016a), where heparin was shown to block the interaction of TAMRA-dsDNA with cells. We have repeated these experiments and confirmed that heparin blocks the interaction of cells with TAMRA-dsDNA. The result obtained indicated that both DNA and heparin compete for the same component of the cell surface. **Thus, the heparin-binding domain(s) of the plasma membrane proteins of TSCs form their general positive charge.** A generalized logical line of results of three independent approaches for the results obtained and described above, namely: 1) direct assessment of the migration of TAMRA+ cells to the cathode; 2) preserving the dsDNA probe-binding mode upon negating the negative charge by positively charged dye Basic Blue GRL 41, and, at the same time; 3) dose-dependent abrogation of the dsDNA probe-binding mode upon negating the positive charge of cells by negatively charged heparin, indicates the presence of a positive charge on cells capable of internalizing the TAMRA-dsDNA probe.

The further logic, as well as its experimental realization were as follows.

4a. There are several groups of factors on the plasma membrane that bind heparin. There are also described three experimentally confirmed heparin binding sites present on the proteoglycans/glycoproteins (PG/GPs), glycosylphosphoinositol-associated proteins (GPI-APs), scavenger receptors (SRs) proteins located on the cell surface and constituting these three groups. The indicated proteins have significantly different linear sizes. PG/GPs can reach up to 1.5 MDa, and is these proteins that compose the basis of glycocalyx, which is well developed in TSCs (Pries et al., 2000; Reitsma et al., 2007; Becker et al., 2010). SR and GPI-AP proteins are 10−30 times smaller than proteoglycans in their linear dimensions. There has appeared a presumption that binding does not always result in internalization, but, probably, these are two in principle independent processes (idea belongs to Yaroslav R. Efremov). The question was raised due to what factors these two processes are going on. **4b-c.** There have been carried out experiments on "cutting" the factors of the cell surface (glycocalyx) using the following proteolytic enzymes: proteinase K (PrK), Trypsin and Collagenase. It has been found that exposure to PrK abrogates the competition

between DNA and heparin for binding sites, as well as massive binding itself that results in significant reduction in fluorescence, but, nevertheless, the internalization of TAMRA-dsDNA into cells retains.

5. Using a set of inhibitors of different types of endocytosis, it has been established that the process of internalization is associated with caveolae and macropinocytosis. Caveolae are featured with clustered SRs and GPI-APs. As it was noted above, PG/GPs are 10−30 times larger than SRs and GPI-APs, and can be located at the different sites of plasma membrane.

6. Comparing the results obtained in paragraphs 5 and 6, we came to the conclusion that PrK hydrolyzes protein components of glycocalyx without reaching the cytoplasmic membrane itself and stops at a certain “height” from the membrane due to steric hindrance caused by glycocalyx structures (sialic acids branching), which limits the depth of hydrolysis. At the same time, the intensity of fluorescence of the labeled material inside a cell has apparently certain limitations, which can not be exceeded even upon excessive superficial binding.

And finally, the results obtained in points 5 and 6 allowed the factors responsible for binding TAMRA-dsDNA and its internalization to be distinguished definitely. Exposure to PrK results in “cutting off” the heparin/DNA-binding sites of PG/GPs, while the DNA-binding sites on SRs and/or GPI-APs remain out of reach of the protease(s). Thus, the heparin/DNA-binding sites on PG/GPs are primarily responsible for the intense fluorescence of TSCs (that allows their distinguishing from the bulk of tumor cells), and the DNA-binding sites on SRs and/or GPI-APs are responsible for the internalization.

7a. The literature analysis conducted suggested that dextran sulfate 500,000 should block all available heparin- and DNA-binding sites, and primarily on SRs (Basu et al., 1979; Harris and Weigel, 2008; Nishinaka et al., 2020). **7b.** It turned out that dextran sulfate 500,000 in fact completely blocks both binding (intense fluorescence of TSCs) and internalization of TAMRA-dsDNA, which confirms the involvement of positively charged domains in the interaction between TSCs and extracellular dsDNA.

The conclusions can be generalized in two following statements.

1. TSCs have a general positive charge, which is determined by the well-developed glycocalyx with an excessive content of heparin-binding domains. This property of TSCs (or any other stem cells) provides the initial, energy-independent aggregation with cells of other types.

2. TSCs appear to have two features associated with interaction with extracellular dsDNA. PG/GPs of the glycocalyx bind dsDNA without being explicitly involved in internalization. It is due to the abundance of these proteins in the glycocalyx and the presence of heparin/DNA-binding

domains in their structure that TAMRA-dsDNA is extensively bound, providing the intense fluorescence, which allows detecting TSCs in the bulk of tumor cells. Internalization of TAMRA-dsDNA into cells occurs as a result of binding to SRs and/or GPI-APs.

Supplementary Material 3 References

Basu, S. K., Brown, M. S., Ho, Y. K., and Goldstein, J. L. (1979). Degradation of low density lipoprotein . dextran sulfate complexes associated with deposition of cholesteryl esters in mouse macrophages. *J. Biol. Chem.* 254, 7141–7146. doi: 10.1016/S0021-9258(18)50296-9.

Becker, B. F., Chappell, D., and Jacob, M. (2010). Endothelial glycocalyx and coronary vascular permeability: The fringe benefit. *Basic Res. Cardiol.* 105, 687–701. doi: 10.1007/S00395-010-0118-Z/FIGURES/2.

Dolgova, E. V., Alyamkina, E. A., Efremov, Y. R., Nikolin, V. P., Popova, N. A., Tyrinova, T. V., et al. (2014). Identification of cancer stem cells and a strategy for their elimination. *Cancer Biol. Ther.* 15, 1378–1394. doi: 10.4161/cbt.29854.

Dolgova, E. V, Petrova, D. D., Proskurina, A. S., Ritter, G. S., Kisaretova, P. E., Potter, E. A., et al. (2019). Identification of the xenograft and its ascendant sphere-forming cell line as belonging to EBV-induced lymphoma, and characterization of the status of sphere-forming cells. *Cancer Cell Int.* 19. doi: 10.1186/s12935-019-0842-x.

Dolgova, E. V, Potter, E. A., Proskurina, A. S., Minkevich, A. M., Chernych, E. R., Ostanin, A. A., et al. (2016a). Properties of internalization factors contributing to the uptake of extracellular DNA into tumor-initiating stem cells of mouse Krebs-2 cell line. *Stem Cell Res. Ther.* 7, 76. doi: 10.1186/s13287-016-0338-8.

Dolgova, E. V, Shevela, E. Y., Tyrinova, T. V, Minkevich, A. M., Proskurina, A. S., Potter, E. A., et al. (2016b). Nonadherent Spheres With Multiple Myeloma Surface Markers Contain Cells that Contribute to Sphere Formation and Are Capable of Internalizing Extracellular Double-Stranded DNA. *Clin. Lymphoma, Myeloma Leuk.* 16, 563–576. doi: 10.1016/j.clml.2016.06.014.

Harris, E. N., and Weigel, P. H. (2008). The ligand-binding profile of HARE: hyaluronan and chondroitin sulfates A, C, and D bind to overlapping sites distinct from the sites for heparin, acetylated low-density lipoprotein, dermatan sulfate, and CS-E. *Glycobiology* 18, 638. doi: 10.1093/GLYCOB/CWN045.

Nishinaka, T., Mori, S., Yamazaki, Y., Niwa, A., Wake, H., Yoshino, T., et al. (2020). A comparative study of sulphated polysaccharide effects on advanced glycation end-product uptake and scavenger receptor class A level in macrophages. *Diabetes Vasc. Dis. Res.* 17. doi: 10.1177/1479164119896975.

Potter, E. A., Dolgova, E. V, Proskurina, A. S., Minkevich, A. M., Efremov, Y. R., Taranov, O. S., et al. (2016). A strategy to eradicate well-developed Krebs-2 ascites in mice. *Oncotarget* 7, 11580–94. doi: 10.18632/oncotarget.7311.

Potter, E. A., Dolgova, E. V, Proskurina, A. S., Zavyalov, E. L., Taranov, O. S., Baiborodin, S. I., et al. (2017). Gene expression profiling of tumor-initiating stem cells from mouse Krebs-2 carcinoma using a novel marker of poorly differentiated cells. *Oncotarget* 8, 9425–9441.

Pries, A. R., Secomb, T. W., and Gaehtgens, P. (2000). The endothelial surface layer. *Pflugers Arch. Eur. J. Physiol.* 440, 653–666. doi: 10.1007/s004240000307.

Reitsma, S., Slaaf, D. W., Vink, H., Van Zandvoort, M. A. M. J., and Oude Egbrink, M. G. A. (2007). The endothelial glycocalyx: composition, functions, and visualization. *Pflugers Arch.* 454, 345. doi: 10.1007/S00424-007-0212-8.
