# Supplementary material for: The new general biological property of stem-like tumor cells Part I. Peculiarities of the process of the double-stranded DNA fragments internalization into stem-like tumor cells
Source: Front Genet. 2022 Sep 8;13:954395. doi: 10.3389/fgene.2022.954395 (PMC9492886; doi:10.3389/fgene.2022.954395)
Supplement: Supplementary file 2 [file DataSheet1.PDF]

Supplementary Material 1

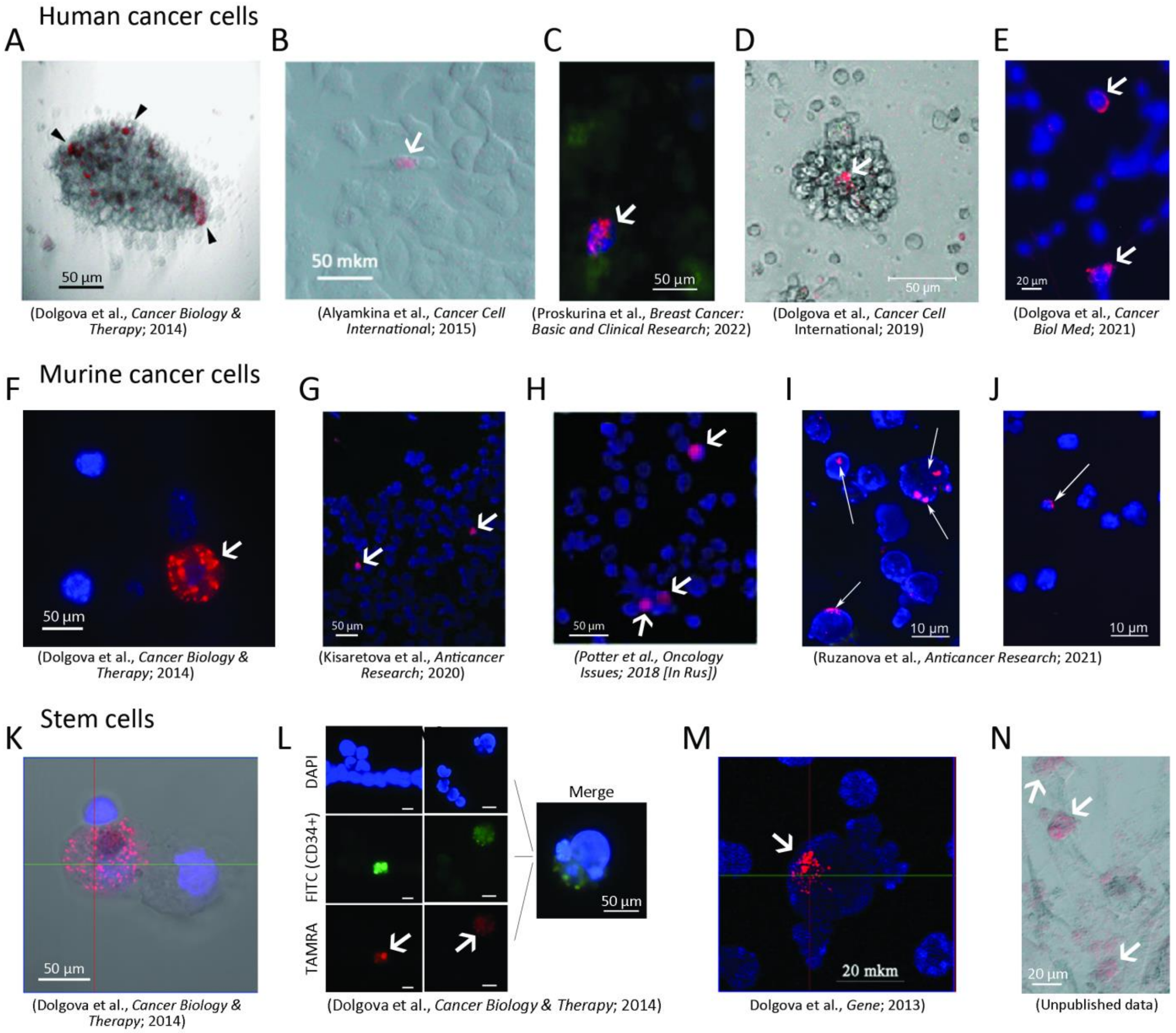

Figure S1-1. Set of images demonstrating that the capability of internalizing extracellular TAMRA-labeled dsDNA fragments is common property for poorly differentiated cells of different origin, both tumor stem-like cells, and normal stem cells. Merged images are shown. Blue (DAPI) – chromatin staining; red (TAMRA) – dsDNA probe staining; white – transmitted light. TAMRA-positive cells are denoted with arrows. (**A**) Primary human glioma neurosphere[1]. (**B**) MCF-7 cells[2]. (**C**) Primary cultures derived from human breast cancer specimens[3]. (**D**) EBV+ B-lymphoma sphere[4]. (**E**) U87 human glioblastoma cells[5]. (**F**) Mouse Krebs-2 carcinoma cells[1]. (**G**) RLS murine lymphosarcoma cells[6]. (**H**) Mouse hepatoma G29 cells[7]. (**I**) Mouse Lewis carcinoma cells[8]. (**J**) Mouse A20 B-cellular lymphoma[8]. (**K**) Human mesenchymal stem cells (shot-term culture)[1]. (**L**) Human hematopoietic stem cells (CD34+ bone marrow cells)[1]. (**M**) Mouse bone marrow cells[9]. (**N**) Human induced pluripotent stem cells culture. A lot of TAMRA+ cells are observed, only some are shown by arrows (not all).

Supplementary Material 1 References

1. Dolgova, E. V *et al.* Identification of cancer stem cells and a strategy for their elimination. *Cancer Biol. Ther.* **15**, 1378–1394 (2014).

2. Alyamkina, E. A. *et al.* Combination of cyclophosphamide and double-stranded DNA demonstrates synergistic toxicity against established xenografts. *Cancer Cell Int.* **15**, (2015).
3. Proskurina, A. S. *et al.* Karanahan: A Potential New Treatment Option for Human Breast Cancer and Its Validation in a Clinical Setting. *Breast Cancer Basic Clin. Res.* **16**, (2022).
4. Dolgova, E. V *et al.* Identification of the xenograft and its ascendant sphere-forming cell line as belonging to EBV-induced lymphoma, and characterization of the status of sphere-forming cells. *Cancer Cell Int.* **19**, (2019).
5. Dolgova, E. V *et al.* Efficacy of the new therapeutic approach in curing malignant neoplasms on the model of human glioblastoma. *Cancer Biol Med* **18**, 910–930 (2021).
6. Kisaretova, P. E. *et al.* Approbation of the cancer treatment approach based on the eradication of TAMRA+ cancer stem cells in a model of murine cyclophosphamide resistant lymphosarcoma. *Anticancer Res.* **40**, 795–805 (2020).
7. Potter, E. A. *et al.* Evaluating the efficiency of the tumor-initiating stem cells eradication strategy on the example of ascite form of mouse gepatocarcinoma G-29. *Oncology Issues* **64**, 818–829 (2018) [In Rus].
8. Ruzanova, V. S. *et al.* Experimental Comparison of the In Vivo Efficacy of Two Novel Anticancer Therapies. *Anticancer Res.* **41**, 3371–3387 (2021).
9. Dolgova, E. V *et al.* Delivery and processing of exogenous double-stranded DNA in mouse CD34 + hematopoietic progenitor cells and their cell cycle changes upon combined treatment with cyclophosphamide and double-stranded DNA. *Gene* **528**, 74–83 (2013).
